# Uncomplexed-TSC1 deploys novel mTORC1-independent pathway to exacerbate the liver glycogen storage in TSC

**DOI:** 10.1038/s41419-025-08161-3

**Published:** 2025-11-14

**Authors:** Xiaoqiao Yue, Yanping Zhang, Na Zhao, Tao Lang, Guangxin Chen, Qiuhong Xiong, Lei Gao, Wenjing Wang, Ping Li, Changxin Wu

**Affiliations:** 1 Institutes of Biomedical Sciences, Shanxi University, Taiyuan, China; 2 Shanxi Provincial Key Laboratory of Medical Molecular Cell Biology, Taiyuan, China; 3 Department of Pathology, The Second Hospital of Shanxi Medical University, Taiyuan, China; 4 School of Medicine, Nankai University, Tianjin, China; 5 Key laboratory of Chemical Biology and Molecular Engineering of Education Ministry, Taiyuan, China

**Keywords:** Mechanisms of disease, Metabolic disorders

## Abstract

Tuberous sclerosis complex (TSC) is an autosomal dominant disorder caused by inactivating mutations in *TSC1* or *TSC2* gene, leading to mTORC1 hyperactivation. However, mTORC1-independent mechanisms in this disorder remain poorly understood. In the study, excess glycogen storage was found in *Tsc1*^–/–^ cells, *Tsc1*^+/–^ and *Tsc1*^*c.2500-2503delAACA*^ mice, as well as in *Tsc2*^–/–^ cells, *Tsc2*^+/^^–^ and *Tsc2*^*c.1113delA*^ mice, with more pronounced accumulation in models with *TSC2* defects. Mechanistically, the deficiency of *TSC1* or *TSC2* gene caused redundant uncomplexed-TSC2 or TSC1 protein, respectively. Strikingly, only uncomplexed-TSC1 downregulated the histone demethylase KDM5A, which in turn increased H3K4me3 levels at the *METTL3* promoter to enhance its expression. The upregulated m^6^A “writer” protein METTL3 cooperated with the “reader” protein IGF2BP2 to stabilize *GYS2* mRNA, causing the upregulation of GYS2 resulting in the glycogen storage. Thus, our study uncovered a novel mTORC1 independent pathway (TSC1-KDM5A-METTL3-IGF2BP2-GYS2) that underlies the excess glycogen storage, and that synergy of mTORC1-dependent and independent pathways leads to the more pronounced glycogen storage with *TSC2* defects compared to those with *TSC1* defects, reflecting the more severer clinical phenotypes in TSC patients with *TSC2* mutations. Importantly, the restoration of glycogen homeostasis and significant amelioration of liver lesion in *TSC2* defect models after the combination treatment of pharmacological inhibitors targeting mTORC1 and METTL3, unveil a potential clinic intervention for TSC patients to whom mTORC1 inhibitors are less effective or even ineffective.

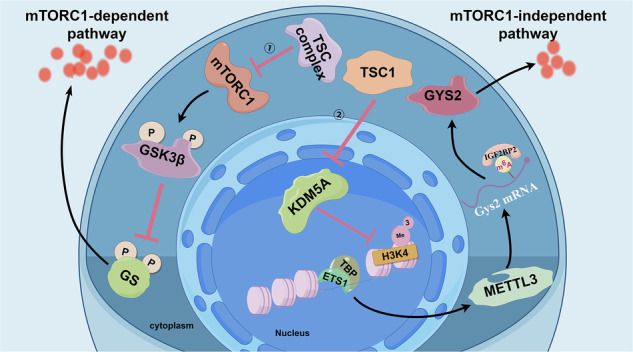

## Introduction

Tuberous sclerosis complex (TSC) is an autosomal dominant disorder caused by inactivating mutations in the *TSC1* or *TSC2* gene [1–3], affecting multiple systems with a wide spectrum of clinical features and disease severity among patients [4]. TSC1 and TSC2 form TSC complex with TBC1D7 that suppresses the activity of the mammalian target of rapamycin complex 1 (mTORC1) [5]. Defect of TSC complex cause the hyperactivation of mTORC1, which is the master regulator of cell growth and metabolism [6, 7]. Currently, the mTORC1 inhibitor everolimus is the primary therapy for TSC. Unfortunately, this may be ineffective and cause severe side effects in some patients [8, 9], suggesting the involvement of mTORC1-independent pathways in TSC pathogenesis. Heterozygous inactivating mutations in *TSC1* or *TSC2* disrupt TSC complex formation, leading to mTORC1 hyperactivation and excess uncomplexed-TSC2 or -TSC1. The effects of excess uncomplexed-TSC1 or -TSC2 in TSC are unknown.

Given that *TSC2* mutations typically result in greater disease severity than *TSC1* mutations [10], we hypothesized that uncomplexed-TSC1 or -TSC2 may have different impacts on TSC disease progression independent of mTORC1. It was known that *TSC1* or *TSC2* gene mutations in TSC patients may cause the neurodegeneration and the development of benign tumors, such as liver or renal angiomyolipomas, renal cell carcinomas, and hamartomas et al. [11–13]. However, research utilizing a mouse model of Lafora disease indicates that glycogen storage directly contributes to neurodegeneration, highlighting glycogen synthesis as a promising target for potential treatment of the condition [14]. Furthermore, glycogen storage played a critical role in tumor initiation. In contrast to advanced tumor cells that consume a large amount of glucose, glycogen accumulation is commonly present in the early stage of human and mouse liver tumors and essential for tumor initiation. Eliminating glycogen accumulation reduces the incidence of liver cancer, whereas increasing glycogen storage accelerates tumorigenesis [15], indicating a potential link between TSC disease progression and glycogen homeostasis.

N6-methyladenosine (m^6^A) methylation modification is one of the most prevalent, abundant, and conserved internal transcriptional modifications of eukaryotic RNA, playing a critical role in gene expression regulation with pivotal roles in diverse physiological and pathological processes [16]. M^6^A methylation is a major modification that occurs mainly in the consensus motif RRm6ACH [17]. Recent studies have reported m^6^A-regulated glucose metabolism-related enzymes to delay or accelerate disease progression. For example, hepatic-specific knockout of METTL3 deteriorates liver I/R injury through reducing the N6-methyladinosine deposition on PCK1 (the rate-limiting enzyme of gluconeogenesis) transcript [18]. However, the role of m^6^A modifications in glycogen metabolism of TSC disease is unknown.

This study investigates the role of uncomplexed-TSC1 on excess glycogen storage mediated by m^6^A modification in TSC disease, unveiling a novel mTORC1-independent pathway: the TSC1-KDM5A-METTL3-IGF2BP2-GYS2 axis. Our findings suggest that targeting this pathway may offer a new therapeutic approach for TSC patients, particularly those with TSC2 mutations.

## Results

### The severity of liver lesions may be correlated with liver glycogen levels in patients

**Fig. 1:**
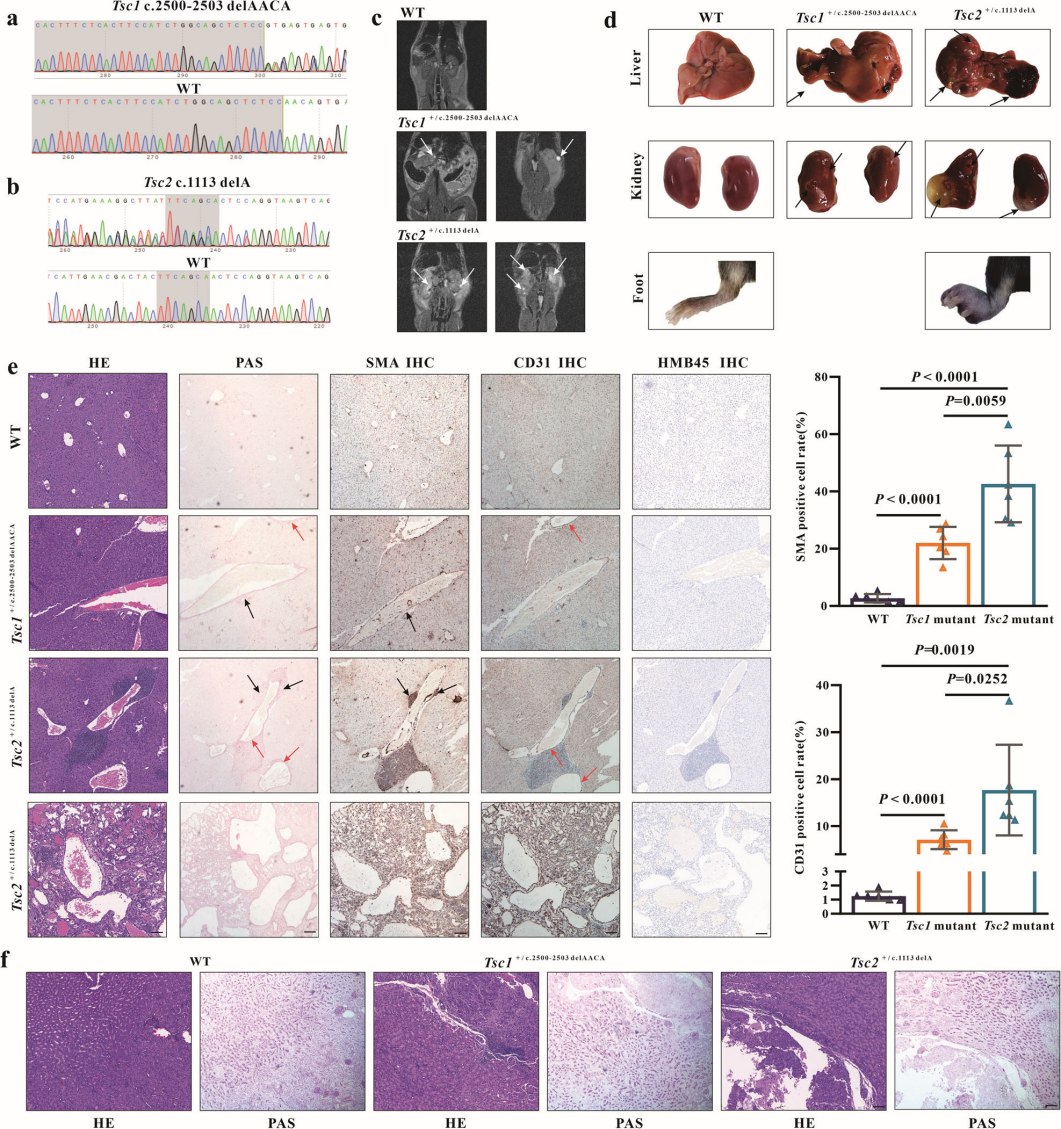
The severity of liver lesions is correlated with glycogen levels in mouse models with *TSC1* or *TSC2* mutations. **a** Confirmation of *Tsc1* mutant mice using Sanger sequencing. **b** Confirmation of *Tsc2* mutant mice using Sanger sequencing. **c** MRI revealed imaging characteristics of the kidneys in 12 to 13 months mice (WT, *Tsc1*^c.2500-2503delAACA^, *Tsc2*^c.1113delA^). **d** Macroscopic appearance of 12 to 13 months mouse tissues (WT, *Tsc1*^c.2500-2503delAACA^, *Tsc2*^c.1113delA^). **e** Representative images of HE staining, SMA IHC staining, CD31 IHC staining, HMB45 IHC staining and PAS staining in liver tissues isolated from mice aged 12 to 13 months (WT, *Tsc1*^c.2500-2503delAACA^, *Tsc2*^c.1113delA −^). *n* = 6 for each genotype; each group contains mice from three different litters. Bar, 100 μm in (**e**). **f** Representative images of HE staining and PAS staining in kidney tissues isolated from mice aged 12 to 13 months (WT, *Tsc1*^c.2500-2503delAACA^, *Tsc2*^c.1113delA^). *n* = 6 for each genotype; each group contains mice from three different litters. Bar, 100 μm in (**f**). Data are shown as the mean ± SD, *n* = 6, two-tailed unpaired Student’s *t*-test.

In our previous study, a known *TSC1* variation c.2509_2512del (p. Asn837Valfs*11, p. N837fs) and a *de novo TSC2* variation c.1113delG (p. Gln371Hisfs*18, p. Q371fs) were identified to affect the formation of the TSC complex [19]. To investigate the causative mechanism in TSC, *Tsc1* heterozygous mutant mice (*Tsc1*^*c.2500-2503delAACA*^) and *Tsc2* heterozygous mutant mice (*Tsc2*^*c.1113delA*^) were generated. Sequencing confirmed the successful establishment of these models (Fig. 1a, b). While survival and development of newborn *Tsc1*^*c.2500-2503delAACA*^ and *Tsc2*^*c.1113delA*^ mice were normal, various tumors emerged in different organs as the animals aged. Macroscopic kidney and liver lesions were observed with a high frequency in *Tsc2*^*c.1113delA*^ mice (60% and 20%) compared to lower frequencies in *Tsc1*^*c.2500-2503delAACA*^ mice (30.8% and 7.7%) (Table 1). Consistent with clinical observations [10], *Tsc2*^*c.1113delA*^ mice exhibited more severe kidney and liver lesions, assessed through Magnetic Resonance Imaging (MRI) (Fig.1c), macroscopic observation (Fig. 1d) and Hematoxylin and eosin (HE) staining (Fig. 1e, f). Additionally, we identified limb lesions (a tumor on the foot) in *Tsc2*^*c.1113delA*^ mice (Fig. 1d).

**Table 1:** Histopathological lesions in mutant mice.

	Tsc1^c.2500-2503delAACA^	Tsc2^c.1113delA^
Age (month)	12–13	12–13
Amount	*n* = 13	*n* = 15
Kidney lesions	4 (30.8%)	9 (60%)
Liver lesions	1 (7.7%)	3 (20%)
Limb lesions	0	1 (6.7%)

In our *Tsc1* and *Tsc2* mutant mice, liver and kidney tumors were observed at the age of 12-month-old. Since the abnormal glycogen accumulation has been linked to tumor development, we examined glycogen content in mouse liver and kidney, and elevated liver glycogen levels were found in all mutant mice. Notably, *Tsc2* mutant mice had significantly higher liver glycogen storage than *Tsc1* mutant mice (Fig. 1e). while kidney glycogen levels remained unchanged (Fig. 1f). The most common liver lesions in TSC patients are hepatic angiomyolipoma which are benign tumors consisting of smooth muscle cells, fibrous tissue, adipose tissue, and abnormally formed vascular channels and are more common in patients with *TSC2* mutations, commonly occurs in adults [20–22]. HMB45 is a hallmark marker for assessing angiomyolipoma [23]. HE and IHC staining with HMB45, SMA (smooth muscle cell marker) and CD31 (vascular endothelial cell marker) were used to evaluate the liver lesion severity and the subtypes. HE staining showed the formation of aberrant highly variable vascular channels. In some regions, the endothelial cell proliferation is quite dense with tiny vascular spaces, although in other regions, relatively large thin-walled vascular channels are seen. IHC showed strong positive expression of CD31 in endothelial cells and SMA in pericytes, while HMB45 was negative, indicating that liver lesions in 12–13-month-old mutant mice were hemangiomas, consisting of proliferative smooth muscle cells, endothelial cells, and vascular channels. *Tsc2* mutant mice had significantly higher CD31 and SMA positive cell rates than *Tsc1* mutant mice. Furthermore, the CD31 and SMA positive areas also exhibited higher glycogen storage (Fig. 1e). These observations suggest that *Tsc1* and *Tsc2* mutant mouse liver hemangioma is a lesion related to human TSC patients and the extent of lesions in TSC patients relates to excess glycogen levels.

### Glycogen storage are increased more pronounced in *TSC2*-deficient cells

**Fig. 2:**
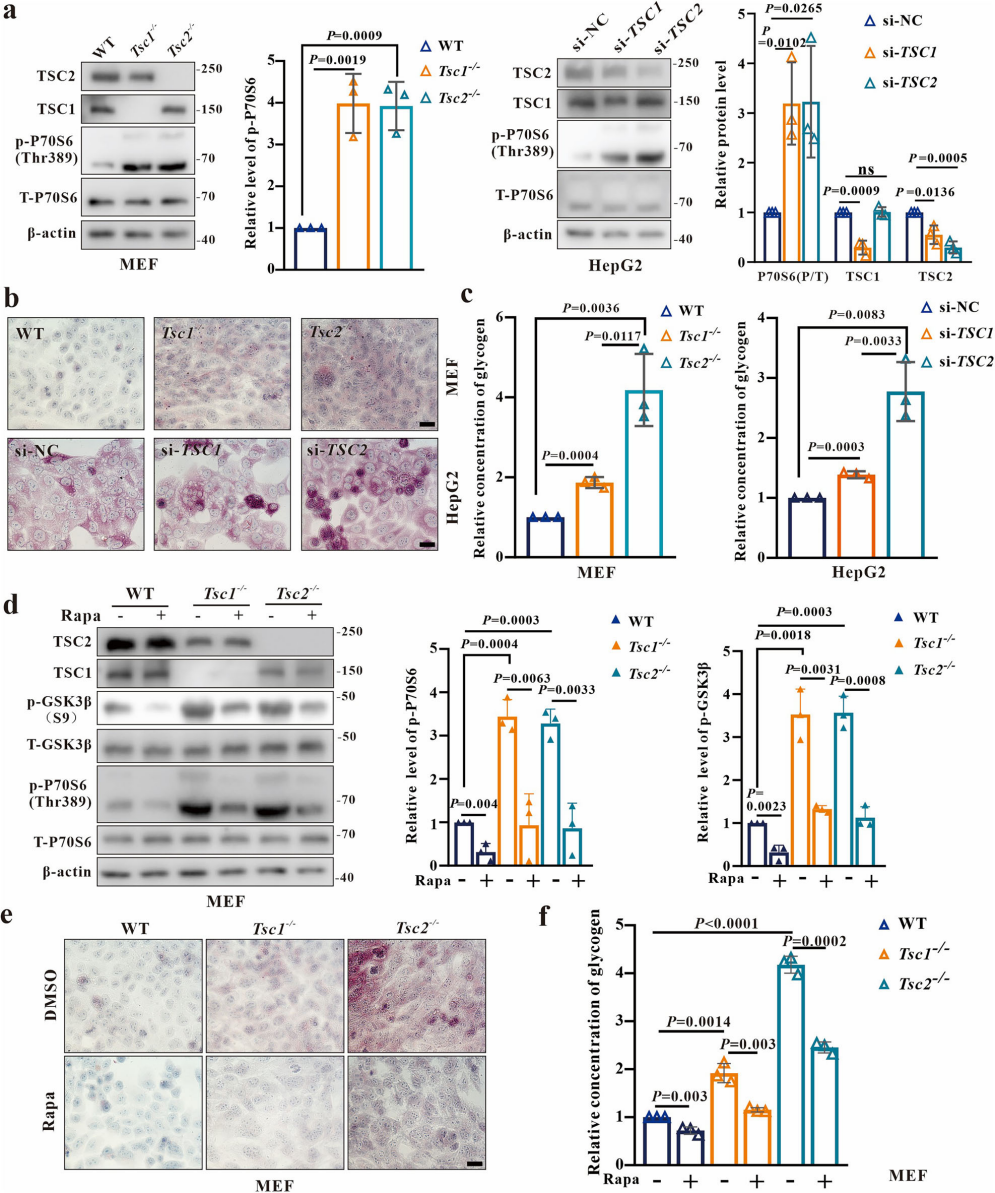
Glycogen storage were significantly increased in *TSC2*-deficient cells compared to *TSC1*-deficient cells. **a** Western blot (WB) analysis of mTORC1 activity in MEFs (WT, *Tsc1*^−/−^, *Tsc2*^−/−^) and HepG2 (si-NC, si-*TSC1*, si-*TSC2*). **b**, **c** Analysis of glycogen levels in MEFs (WT, *Tsc1*^−/−^, *Tsc2*^−/−^) and HepG2 (si-NC, si-*TSC1*, si-*TSC2*) using Periodic Acid-Schiff (PAS) staining and a glycogen assay kit. Bar, 20 μm in (**b**). **d** Detection of mTORC1 activity and p-GSK3β(s9) levels in MEFs (WT, *Tsc1*^−/−^, *Tsc2*^−/−^) using WB at 24 h post treatment of 300 nM rapamycin. **e**, **f** Analysis of glycogen levels in MEFs (WT, *Tsc1*^−/−^, *Tsc2*^−/−^) at 24 h post treatment of 300 nM rapamycin or control (DMSO) using PAS staining and glycogen assay kit. Bar, 20 μm in (**e**). Data are shown as the mean ± SD, *n* = 3, two-tailed unpaired Student’s *t*-test.

To investigate the mechanism behind increased glycogen levels in TSC, mTORC1 activity in cellular models was analyzed. Results indicated a remarkable increase in basal mTORC1 activity, as evidenced by elevated p-P70S6(Thr389). Furthermore, HepG2 cells treated with *TSC1* siRNA (HepG2-si*TSC1*) and *TSC2* siRNA (HepG2-si*TSC2*) also showed increased mTORC1 activity (Fig. 2a). To determine whether the absence of TSC1 or TSC2 affects cellular glycogen levels, quantitative measurement of glycogen was conducted. Periodic Acid-Schiff (PAS) staining and glycogen assays demonstrated elevated glycogen levels in all knockout (KO) and knockdown (KD) cells (Fig. 2b, c).

It is known that the activation of the mTORC1-GSK3β axis increased the gluconeogenesis in *Tsc2*^−/−^ MEFs [24], but the role of it in *Tsc1*^−/−^ MEFs is still unknown. The results showed that p-GSK3β(S9) levels in *Tsc1*^−/−^ and *Tsc2*^−/−^MEFs were all increased, and were partially reduced compared to untreated cells in rapamycin (first-generation mTORC1 inhibitor) treated MEFs (Fig. 2d). We noticed that excess glycogen storage in *TSC2*-deficient cells were increased more pronounced than in *TSC1*-deficient cells, consistent with in vivo results. Rapamycin and AZD-8055 (a second-generation mTORC1 inhibitor) treatment restored the glycogen levels in *Tsc1*^−/−^ MEFs similar to those in wild-type (WT) MEFs, while the glycogen levels in *Tsc2*^−/−^ MEFs did not fully return to normal (Figs. 2e, f, and S1a-b), indicating mTORC1-independent pathways may manipulate glycogen levels in *TSC2*-deficient cells.

### The m^6^A modification is increased upon *TSC1* and *TSC2* depletion

**Fig. 3:**
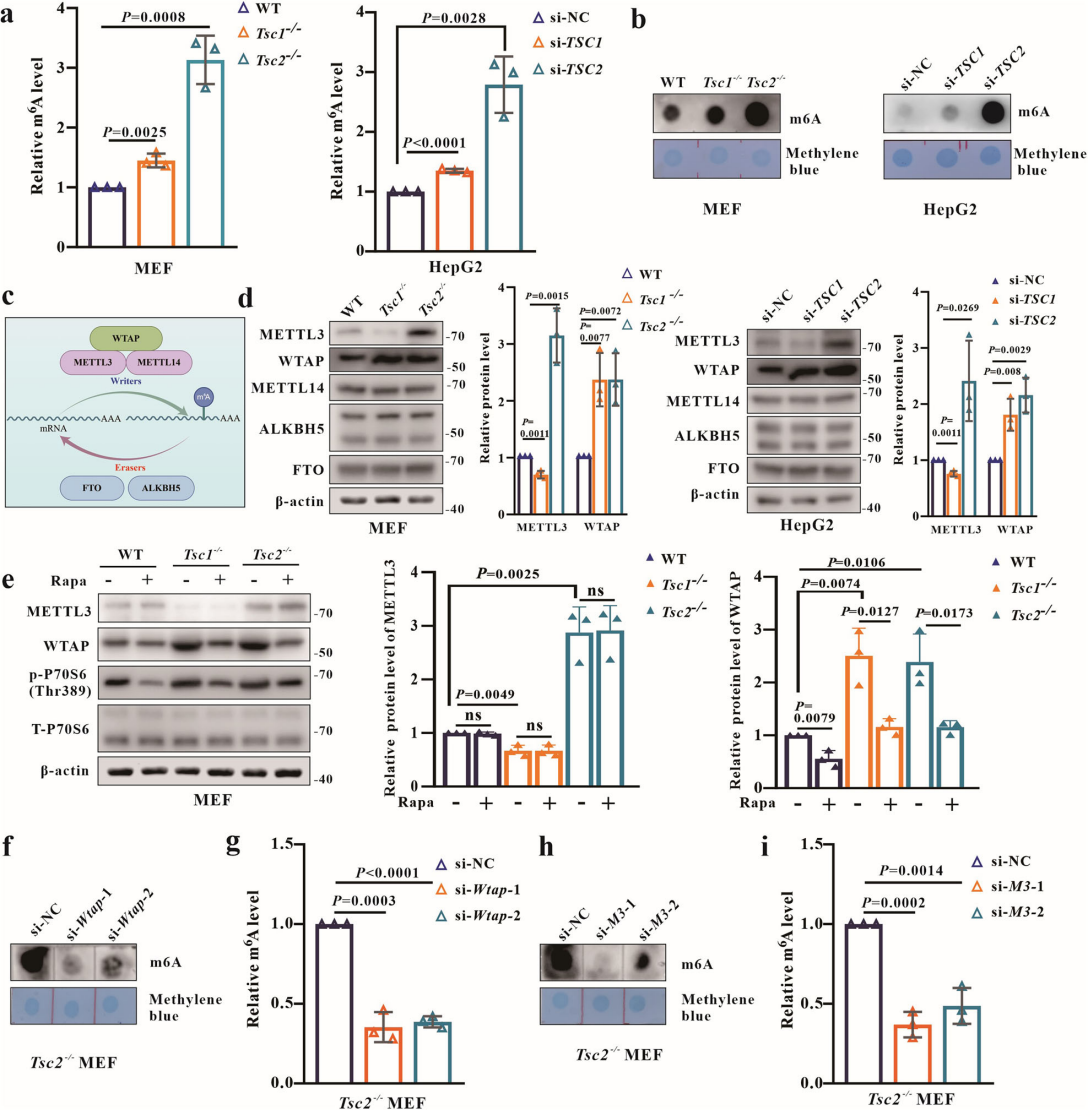
The m^6^A modification is altered upon depletion of *TSC1* and *TSC2.* **a**, **b** Detection of m^6^A levels in MEFs (WT, *Tsc1*^−/−^, *Tsc2*^−/−^) and HepG2 (si-NC, si-*TSC1*, si-*TSC2*) using an m^6^A quantification assay and m^6^A dot blot. **c** The main enzymes involved in m^6^A modification, created with Figdraw. **d** WB analysis of METTL3, METTL14, WTAP, FTO and ALKBH5 expression in MEFs (WT, *Tsc1*^−/−^, *Tsc2*^−/−^) and HepG2 (si-NC, si-*TSC1*, si-*TSC2*). **e** WB analysis of the expression of WTAP, METTL3 and p-P70S6 (Thr389) in MEFs (WT, *Tsc1*^−/−^, *Tsc2*^−/−^) at 24 h post-treatment with 300 nM rapamycin or DMSO. **f**, **g** Detection of m^6^A levels in *Tsc2*^−/−^ MEFs (si-NC, si-*Wtap-*1, si-*Wtap-*2) using m^6^A quantification assay and m^6^A dot blot. **h**, **i** Detection of m^6^A levels in *Tsc2*^−/−^ MEFs (si-NC, si-*Mettl3-*1, si-*Mettl3-*2) using m^6^A quantification assay and m^6^A dot blot. Data are shown as the mean ± SD, *n* = 3, two-tailed unpaired Student’s *t*-test.

M^6^A modification has been shown to regulate glucose metabolism through various pathways, including PI3K–AKT–mTORC1 [25], Wnt/β-catenin [26], and RhoA/ROCK signaling pathways [27], but the implications of m^6^A modification in glycogen storage in TSC have not been investigated. The m^6^A levels were measured and the results demonstrated the increased m^6^A modification in both *Tsc1*^−/−^ and *Tsc2*^−/−^ MEFs, with a more significant increase in *Tsc2*^−/−^ MEFs (Fig. 3a). Similar results were observed in HepG2-si*TSC1* and HepG2-si*TSC2* cells (Fig. 3a, b). The expressions of the m^6^A methyltransferase complex (WTAP-METTL3-METTL14) and demethylases (FTO and ALKBH5) were measured for further confirmation (Fig. 3c). The WTAP expressions were upregulated in all KO MEFs and KD HepG2 cells. Notably, the METTL3 expression was decreased in *TSC1* KD/KO cells but increased in *TSC2* KD/KO cells (Fig. 3d).

To determine whether the changes in WTAP and METTL3 were caused by mTORC1 pathway hyperactivation, all MEFs were treated with rapamycin. Surprisingly, the expression of WTAP was decreased, while the expression of METTL3 remained unchanged after drug treatment (Fig. 3e). Further, the m^6^A levels were detected in *Tsc2*^−/−^ MEFs with *Wtap* or *Mettl3* knockdown. The results showed that the increased m^6^A level from *Tsc2*^−/−^ MEFs was reduced upon *Wtap* or *Mettl3* knockdown (Figs. 3f–i, and S2a, c). These results imply that WTAP and METTL3 mediate the m^6^A modification in TSC, and the WTAP expression rather than METTL3 was regulated by mTORC1.

### The upregulation of METTL3 increases the glycogen storage in HepG2 cells

**Fig. 4:**
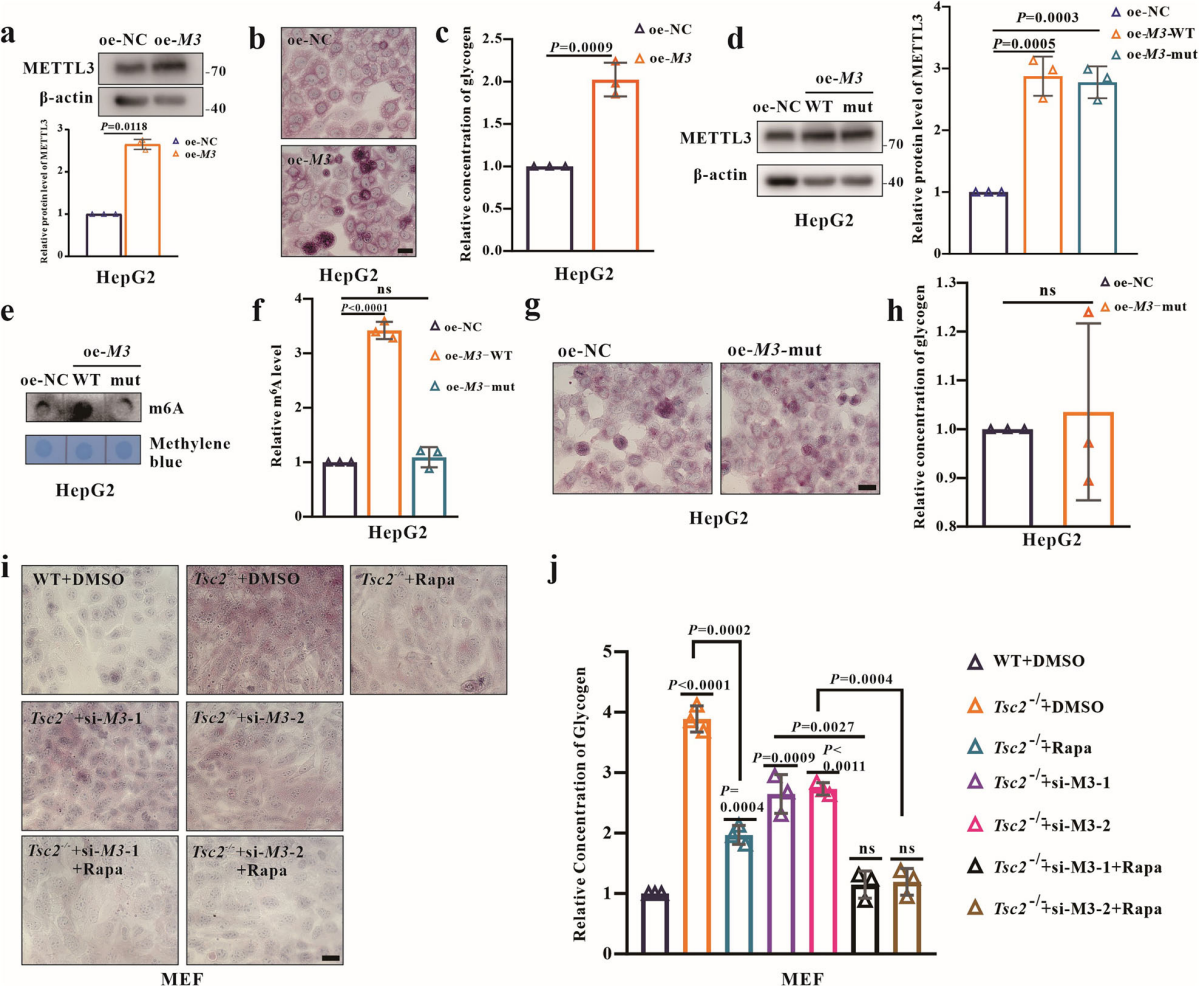
METTL3 affects the glycogen level in cells. **a** Detection of transient expression of *METTL3* in HepG2 using WB at 48 h post-transfection with wild-type *METTL3* (oe-*M3*). **b**, **c** Analysis of glycogen levels in HepG2 (oe-NC, oe-*M3*) using PAS staining and the glycogen assay kit. Bar, 20 μm in (**b**). **d** Detection of *METTL3* transient expression in HepG2 using WB at 48 h post transfection with wild type METTL3 (oe-*M3-*WT) and the catalytically dead mutant of METTL3 (mut *METTL3*; residues 395–398: DPPW→APPA) (oe-*M3*-Mut). **e**, **f** Detection of m^6^A levels in HepG2 (oe-NC, oe-*M3*-WT, oe-*M3*-Mut) using m^6^A dot blot and m^6^A quantification assay. **g**, **h** Analysis of glycogen levels in HepG2 (oe-NC, oe-*M3*-Mut) using PAS staining and the glycogen assay kit. Bar, 20 μm in (**g**). **i**, **j** Detection of glycogen levels at 24 h post-treatment with rapamycin (300 nM), or DMSO in MEFs transfected with si-NC, si-*M3*-1, si-*M3*-2 for 48 h. Bar, 20 μm in (**i**). Data are shown as the mean ± SD, *n* = 3, two-tailed unpaired Student’s *t*-test.

Given the altered expressions of METTL3 and WTAP in *TSC1* and *TSC2*-deficient cells, we investigated their association with excess glycogen storage. *METTL3* was overexpressed in HepG2 (oe-*M3*) cells (Fig. 4a) and glycogen levels were quantified, finding that *METTL3* overexpression increased glycogen levels (Fig. 4b, c). Then *WTAP* were knocked down in *Tsc1*^−/−^, *Tsc2*^−/−^ MEFs and HepG2 cells; however, the depletion of WTAP had no significant effect on glycogen levels (Fig. S2b–g). Moreover, METTL3 has been reported to play important roles in both m^6^A-dependent and -independent manners [28, 29]. To clarify whether METTL3 affects glycogen storage in an m^6^A-dependent or independent manner, a plasmid containing catalytically inhibited *METTL3* (aa395-398, DPPW→APPA) (oe-*M3*-mut) was constructed to assess its activity in relation to glycogen storage. In *METTL3*-WT overexpression cells, the glycogen levels were increased (Fig. 4b, c). However, in *METTL3*-mut overexpression cells, the glycogen levels and m^6^A methylation enzyme activity were similar to those in control cells (Fig. 4d–h), indicating that METTL3 regulates glycogen storage in an m^6^A-dependent manner. To determine if glycogen storage in TSC is regulated by both METTL3 and mTORC1, *METTL3*-siRNA transfection and/or rapamycin treatment were performed. PAS staining and glycogen assays showed that either treatment partially decreased glycogen levels in *Tsc2*^−/−^ MEFs, while the combined treatment dramatically reduced excess glycogen to levels comparable to WT MEFs (Fig. 4i, j). Overall, these results indicate that aberrant METTL3 expression in *TSC1* or *TSC2*-deficient cells manipulates glycogen storage via an mTORC1-independent pathway, while WTAP may play other roles in TSC pathogenesis.

### Redundant uncomplexed-TSC1 increases glycogen storage by upregulating the expression of METTL3

**Fig. 5:**
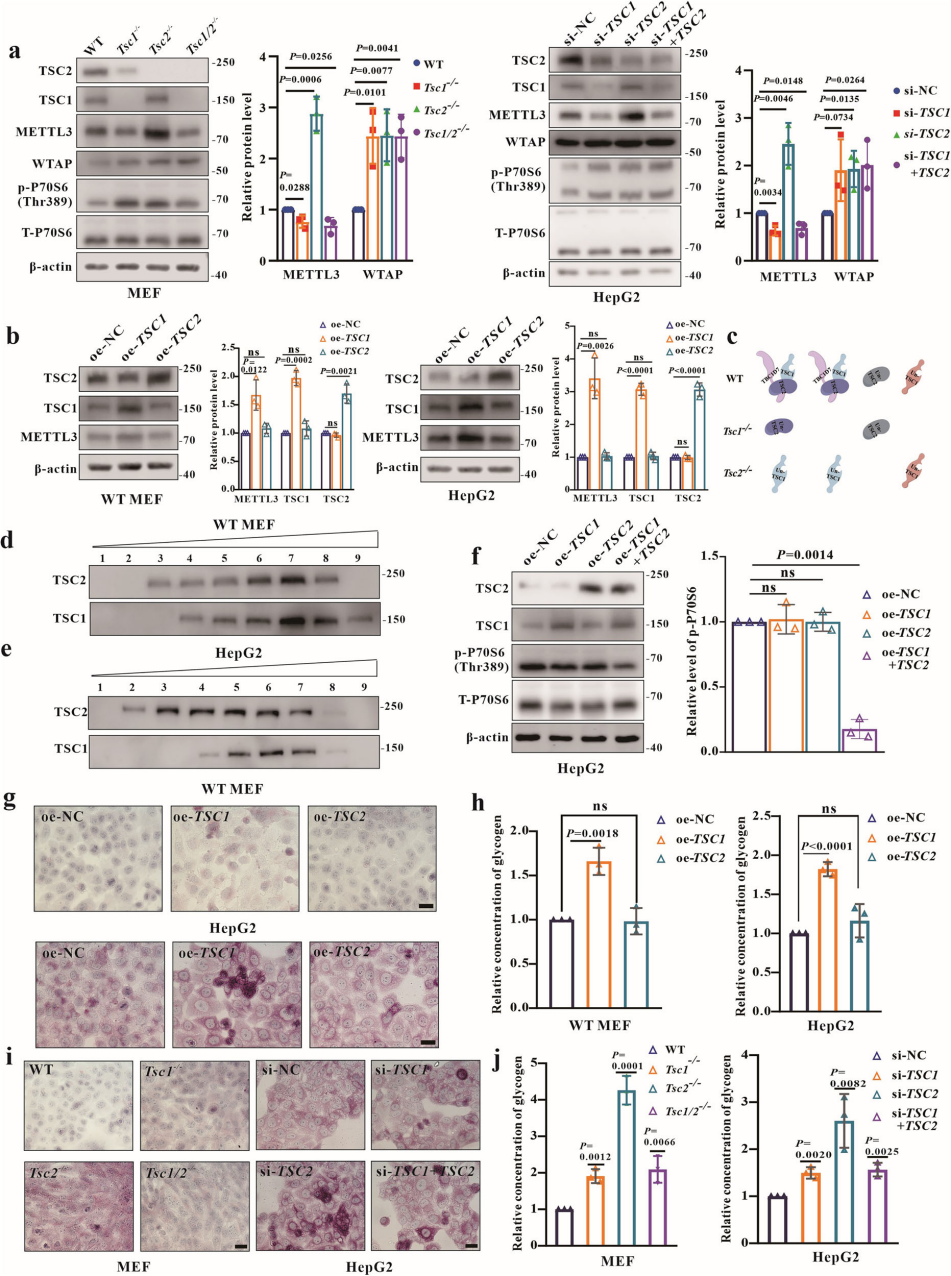
Redundant uncomplexed-TSC1 increases glycogen storage by upregulating METTL3. **a** WB analysis of mTORC1 activity, METTL3 and WTAP protein levels in MEFs (WT, *Tsc1*^−/−^, *Tsc2*^−/−^, *Tsc1/2*^−/−^) and HepG2 (si-NC, si-*TSC1*, si-*TSC2*, si-*TSC1*+*TSC2*). **b** Detection of METTL3 expression in WT MEFs and HepG2 with transient overexpression of *TSC1* or *TSC2*. **c** Schematic representation of the presence of TSC1, TSC2, and TSC complex forms in four types of MEFs, created using Figdraw. *TSC1* knockout (*Tsc1*^–/–^ MEFs) causes loss of TSC1 and increased expression of uncomplexed-TSC2, due to loss of TSC1 leads to reductions of TSC2 at the same time, only increased a part of expression of uncomplexed-TSC2. *TSC2* knockout (*Tsc2*^–/–^ MEFs) causes the loss of TSC2 and increased uncomplexed-TSC1 level, double knockout (*Tsc1/2*^–/–^ MEFs) caused loss of TSC1 and TSC2. **d**, **e** Analysis of TSC1, TSC2 and TSC complex in samples prepared from WT MEFs and HepG2 post **s**ucrose density-gradient centrifugation using WB (fractions 1 to 9 were arranged from top to bottom). **f** Analysis of mTORC1 activity in HepG2 with transient overexpression of *TSC1* or *TSC2*. **g**, **h** Analysis of glycogen levels in MEFs and HepG2 using PAS staining and the glycogen assay kit at 48 h post transient overexpression of *TSC1* or *TSC2*. Bar, 20 μm in (**g**). **i**, **j** Quantification of glycogen levels in MEFs (WT, *Tsc1*^−/−^, *Tsc2*^−/−^, *Tsc1/2*^−/−^) and HepG2 (si-NC, si-*TSC1*, si-*TSC2*, si-*TSC1*+*TSC2*). Bar, 20 μm in (**i**). Data are shown as the mean ± SD, *n* = 3, two-tailed unpaired Student’s *t*-test.

We observed that the expression of METTL3 was decreased in *Tsc1*^–/–^ MEFs but increased in *Tsc2*^–/^^–^ MEFs. Therefore, the double knockout MEFs (*Tsc1*/2^–/–^) were generated for further study (Figs. 5a, and S3a–c). The hyperactivation level of mTORC1 was similar in all KO and KD cells. Subsequently, the METTL3 expression measurement assay showed that the METTL3 expression in both *Tsc1*/2^–/–^ MEFs and HepG2-si*TSC1/2* was reduced to similar levels detected from *Tsc1*^–/–^ MEFs and HepG2-si*TSC1* (Fig. 5a).

To understand why METTL3 was decreased in *Tsc1*^–/–^ and *Tsc1/2*^–/–^ MEFs but increased in *Tsc2*^–/–^ MEFs, we hypothesized that both TSC1 and TSC2 regulate METTL3 expression independent of the TSC complex. Overexpressing *TSC1* or *TSC2* in both HepG2 and MEFs demonstrated that only *TSC1* overexpression could lead to the increased expression of METTL3 (Fig. 5b), indicating that redundant uncomplexed-TSC1 (caused by *TSC2* defects or *TSC1* overexpression) downregulates the expression of METTL3 (Fig. 5b, c, Table 2).

**Table 2:** Analysis of the various forms of TSC1, TSC2 and TSC complex in all MEFs.

	WT	Tsc1^–/–^	Tsc2^–/^^–^	Tsc1/2^–/–^
TSC complex	**−**	↓	↓	↓
TSC1(All forms)	**−**	↓	−	↓
Uncomplexed-TSC1	**−**	↓	↑	↓
TSC2(All forms)	**−**	↓	↓	↓
Uncomplexed-TSC2	**−**	↑	↓	↓

Sucrose density gradient assays confirmed the presence of complexed and uncomplexed TSC1 and TSC2 in WT MEFs and HepG2 cells (Fig. 5d, e). It remains unclear whether overexpression of *TSC1* or *TSC2* affects mTORC1 activity. We examined p-P70S6(Thr389) levels in cells with *TSC1* and *TSC2* overexpression, revealing that co-overexpression of *TSC1* and *TSC2* inhibited mTORC1 activity, while single overexpression of either did not (Fig. 5f). These results demonstrate that uncomplexed-TSC1 mediates METTL3 expression in an mTORC1-independent manner.

To clarify whether uncomplexed-TSC1 impacts glycogen storage in TSC, PAS staining and glycogen assays were conducted in all *TSC1* or *TSC2* overexpression cells, showing that *TSC1* overexpression boosted glycogen storage, while *TSC2* did not (Fig. 5g, h). Further investigations revealed consistent glycogen storage trends in *TSC1* KO/KD and *TSC1/2* KO/KD cells (Fig. 5i, j). These results indicate that uncomplexed-TSC1 increases glycogen storage mediated by METTL3 through a novel TSC1-METTL3-mTORC1-independent pathway.

### Redundant uncomplexed-TSC1 activates METTL3 transcription by KDM5A-H3K4me3-*METTL3* promoter axis

**Fig. 6:**
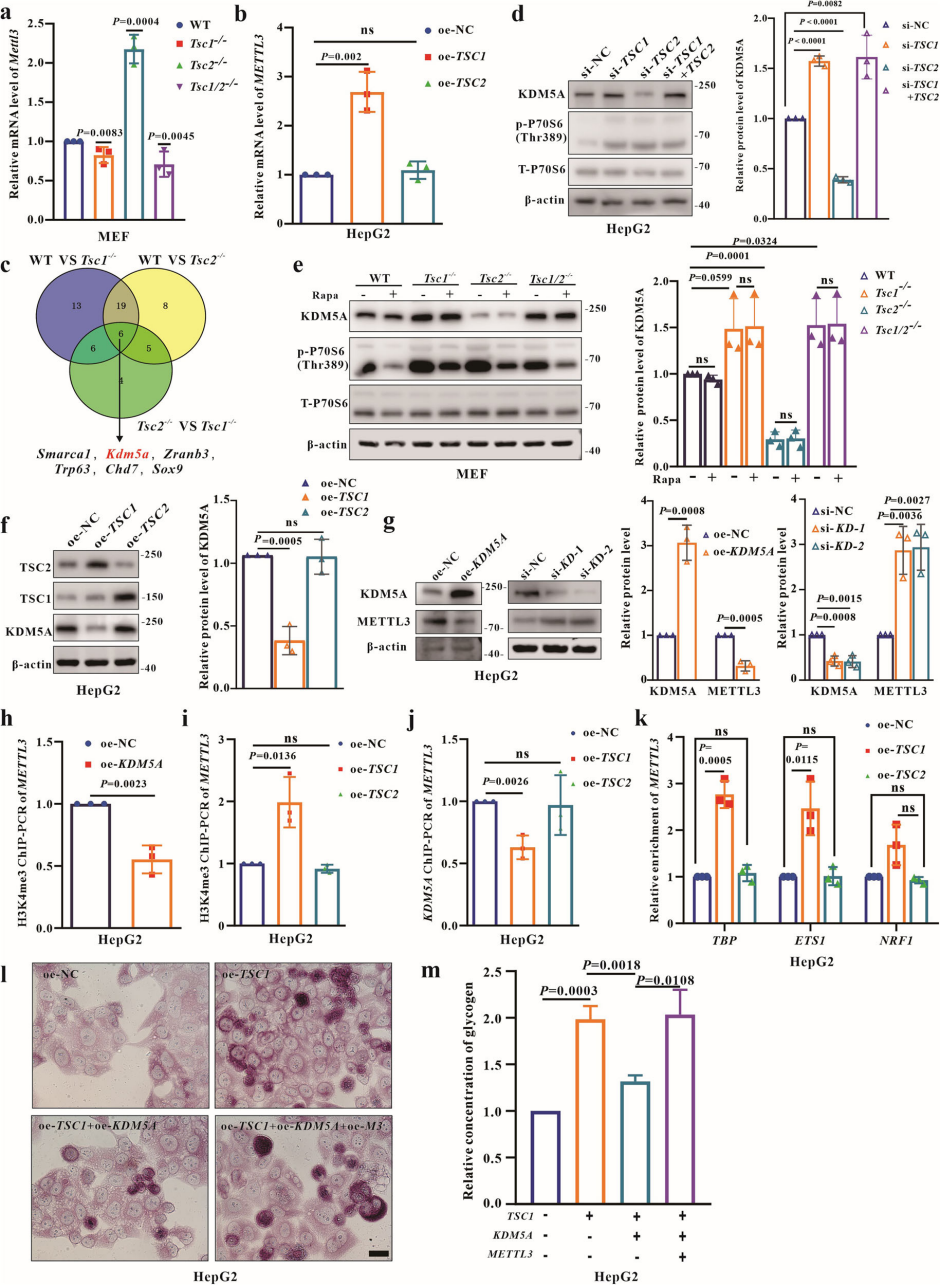
Redundant uncomplexed-TSC1 activates the transcription of *METTL3* by inhibiting KDM5A-mediated H3K4me3 demethylation modification of the *METTL3* promoter. **a** Analysis of *Mettl3* mRNA expression in all MEFs using qRT-PCR. **b** Analysis of *METTL3* mRNA expression in oe-NC, oe-*TSC1* and oe-*TSC2* HepG2 using qRT-PCR. **c** Venn diagram showing overlaps between differential genes of three RNA-seq with GO molecular function including chromatin remodeling. **d** WB analysis of KDM5A in HepG2 (si-NC, si-*TSC1*, si-*TSC2*, si-*TSC1*+*TSC2*). **e** Analysis of KDM5A expression in MEFs (WT, *Tsc1*^−/−^, *Tsc*2^−/−^, *Tsc1/2*^−/−^) at 24 h post treatment of 300 nM rapamycin or DMSO. **f** Analysis of KDM5A expression in oe-NC, oe-*TSC1* and oe-*TSC2* HepG2 using WB. **g** Detection of KDM5A and METTL3 in HepG2 at 48 h post transient overexpression or knockdown of *KDM5A* by transfection with overexpressing vector (oe-*KDM5A*) or *KDM5A*-siRNA (si-*KDM5A*-1, si-*KDM5A*-2). **h** ChIP-PCR analysis of H3K4me3 modification within the *METTL3* promoter in oe-NC and oe-*KDM5A* HepG2. **i** ChIP-PCR analysis of H3K4me3 modification within the *METTL3* promoter in oe-NC, oe-*TSC1* and oe-*TSC2* HepG2. **j** ChIP-PCR analysis of KDM5A binding with *METTL3* promoter in oe-NC, oe-*TSC1* and oe-*TSC2* HepG2. **k** ChIP-PCR analysis of TFs TBP, ETS1 and NRF1 binding with *METTL3* promoter in oe-NC, oe-*TSC1* and oe-*TSC2* HepG2. **l**, **m** Detection of glycogen levels in HepG2 using PAS staining and the glycogen assay kit at 48 h post transfection with *TSC1* overexpressing vector, *TSC1* overexpressing vector + *KDM5A* overexpressing vector, or *TSC1* overexpressing vector + *KDM5A* overexpressing vector + *METTL3* overexpressing vector. Bar, 20 μm in (**l**). Data are shown as the mean ± SD, *n* = 3, two-tailed unpaired Student’s *t*-test.

To clarify how uncomplexed-TSC1 mediates METTL3 hyperexpression and whether it is due to the increased transcription or enhanced stability, a systematic series of experiments were performed. The half-life of METTL3 protein in WT and *Tsc2*^–/^^–^ MEFs showed no significant difference after cycloheximide (CHX) treatment (Fig. S4a, b), suggesting uncomplexed-TSC1 regulates METTL3 synthesis rather than stability. Analyzing *METTL3* mRNA expression via RT-qPCR showed consistent changes in mRNA and protein levels (Fig. 6a, b), indicating uncomplexed-TSC1 is involved in regulating *METTL3* mRNA expression. To assess if mRNA expression changes were due to increased transcription or stability, the half-life of endogenous *METTL3* mRNA in WT and *Tsc2*^–/–^ MEFs after actinomycin D (Act-D) treatment was tested. The half-life of *METTL3* mRNA showed no significant alteration (Fig. S4c), suggesting uncomplexed-TSC1 may induce *METTL3* transcription hyperactivation. TBP, NRF1, and ETS1 are known transcription factors (TFs) involved in *METTL3* transcription [30, 31]. The expression levels of these TFs in *Tsc2*^–/–^ and oe-*Tsc1* MEFs were measured. Both the mRNA and protein levels of these TFs remained unchanged (Fig. S5a, b). It was reported that ETS1 translocate to the cytoplasm in response to calcium-induced signals [32], however, ETS1 nuclear relocation was not observed in cells with *TSC1* overexpression (Fig. S5c).

Chromatin remodeling affects TFs binding within promoter regions [33]. Based on sequencing results, six candidates differentially expressed genes (DEGs) were predicted from three RNA-seq datasets, including *Smarca1* located on the X chromosome. The screening criteria for DEGs included Gene Ontology (GO) molecular functions related to chromatin remodeling (GO:0006338) (Fig. 6c). Quantification of RNA expression of five candidate DEGs showed that *KDM5A* and *SOX9* were downregulated in *TSC1* overexpressing cells (Fig. S5d). KDM5A is a histone demethylase that removes H3K4me3/me2 [34]. It is known that abundant H3K4me3 in the *METTL3* promoter positively regulates global transcription activity [35]. Therefore, we wondered whether KDM5A modulates the transcription of *METTL3* independent of mTORC1 in TSC. Interestingly, WB experiments revealed that KDM5A was decreased in both *TSC2* KO/KD cells and *TSC1* overexpressing cells, but increased in both *TSC1* KO/KD and *TSC1/2* KO/KD cells (Fig. 6d–f). In contrast, KDM5A expressions were unchanged in *TSC2* overexpressing cells (Fig. 6f), and unaffected upon rapamycin treatment in MEFs (Fig. 6e). Furthermore, KDM5A negatively regulated METTL3 expression based on knockdown and overexpression studies (Fig. 6g). Accordingly, chromatin immunoprecipitation (ChIP) assays were conducted to quantify H3K4me3 level. The ChIP-PCR results showed that *KDM5A/TSC1* overexpression induced lower/higher H3K4me3 modification in the *METTL3* promoter (Fig. 6h, i). Moreover, less KDM5A bound to the *METTL3* promoter under *TSC1* overexpression condition (Fig. 6j). Next, we tested whether uncomplexed-TSC1 controls the binding strength of TFs within the *METTL3* promoter region. ChIP assays revealed that TBP and ETS1 binding to the *METTL3* promoter increased with *TSC1* overexpression (Fig. 6k). Finally, PAS staining and glycogen assays showed that overexpressed *KDM5A* reduced excess glycogen storage induced by *TSC1* overexpression, while overexpressed *METTL3* increased glycogen storage under *KDM5A* overexpression in HepG2 cells (Fig. 6l, m). The results indicate that uncomplexed-TSC1 activates *METTL3* transcription by inhibiting KDM5A-mediated H3K4me3 demethylation in the *METTL3* promoter.

### GYS2 as a key target of METTL3 regulates glycogen storage in an IGF2BP2-dependent manner in TSC

**Fig. 7:**
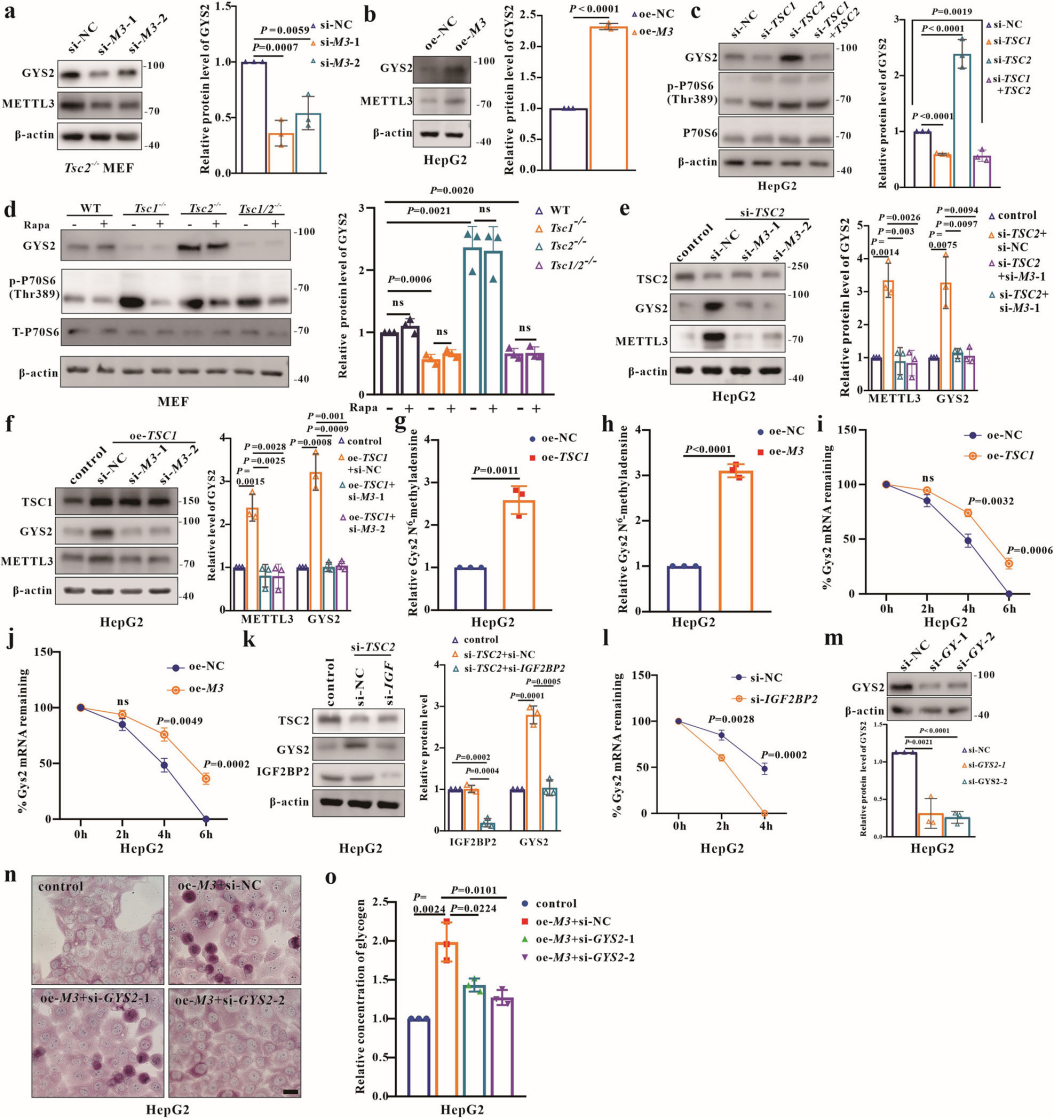
GYS2 is a responsive target of METTL3 in TSC. **a** WB analysis of METTL3 and GYS2 in *Tsc2*^−/−^ MEFs (si-*M3*-1, si-*M3*-2*)*. **b** WB analysis of METTL3 and GYS2 in oe-NC and oe-*M3* HepG2. **c** WB analysis of GYS2 in HepG2 (si-NC, si-*TSC1*, si-*TSC2*, si-*TSC1*+*TSC2*). **d** Analysis of GYS2 in MEFs (WT, *Tsc1*^−/−^, *Tsc*2^−/−^, *Tsc1/2*^−/−^) at 24 h post-treatment with 300 nM rapamycin or DMSO using WB. **e**, **f** Detection of METTL3 and GYS2 in HepG2 at 48 h post the transfection of si-*TSC2* or oe-*TSC1*, and si-*METTL3*. **g** M^6^A methylation detection of *GYS2* in oe-NC and oe-*TSC1* HepG2. **h** M^6^A methylation detection of *GYS2* in oe-NC and oe-*M3* HepG2. **i** Detection of the mRNA half-life of *GYS2* in oe-NC and oe-*TSC1* HepG2. **j** Detection of the half-life of mRNA *GYS2* in oe-NC and oe-*M3* HepG2. **k** Detection of IGF2BP2 and GYS2 in HepG2 at 48 h post the transfection of si-*TSC2* and si-*IGF2BP2*. **l** Detection of the mRNA half-life of *GYS2* in si-NC and si- *IGF2BP2*. **m** WB quantification of knockdown efficacy of *GYS2* in HepG2 (si-*GYS2*-1, si-*GYS2*-2 targeting human *GYS2*). **n**, **o** Analysis of glycogen levels in HepG2 at 48 h post transfection of oe-*M3* and si-*GYS2*; Bar, 20 μm in (**n**). Data are shown as the mean ± SD, *n* = 3, two-tailed unpaired Student’s *t*-test.

To identify how METTL3 regulates glycogen storage in TSC, the mRNA expressions of the glycogen synthesis genes were analyzed. Results indicated that only *GYS2* mRNA expression was upregulated in *METTL3*-overexpressing cells (Fig. S6a). To confirm whether GYS2 is a responsive target of METTL3 in TSC, the expression of GYS2 was examined in cells with *METTL3* knockdown or overexpression, respectively. GYS2 was repressed in *METTL3* knockdown cells (Fig. 7a) and upregulated in *METTL3* overexpressing cells (Fig. 7b), demonstrating that GYS2 is a downstream target of METTL3 in TSC. Meanwhile, the GYS2 expression was consistent with the METTL3 expression in cells with *TSC1* or *TSC2* defect or overexpression, respectively (Figs. 7c–f, and S6b). And GYS2 protein levels remained unchanged after rapamycin treatment (Fig. 7d), indicating GYS2 expression modulation is mTORC1-independent. Furthermore, the increased GYS2 expression in *TSC2*-deficient or *TSC1*-overexpressing cells was reduced by *METTL3* knockdown (Fig. 7e, f). These results reveal that the uncomplexed-TSC1-METTL3 axis mediates GYS2 expression in TSC. To clarify whether *GYS2* mRNA is a methylation substrate of METTL3 in TSC, methylated RNA immunoprecipitation followed by quantitative PCR (MeRIP-qPCR) was performed. The results showed that the m^6^A modification of *GYS2* mRNA were activated in both *TSC1* or *METTL3* overexpressed cells (Fig. 7g, h). The half-life of endogenous *GYS2* mRNA was determined and the results showed that it degraded more slowly when *TSC1* or *METTL3* was overexpressed in HepG2 cells (Fig. 7i, j). It has been reported that N6-methyladenosine stabilizes *GYS2* mRNA in an IGF2BP2-dependent manner [36]. Thus, we tested the protein expression of GYS2 in *IGF2BP2* and *TSC2* knockdown cells, and found that the increased GYS2 protein expression in *TSC2*-deficient cells was reduced upon *IGF2BP2* knockdown (Fig. 7k). Furthermore, mRNA decay assays demonstrated a downregulation of IGF2BP2 expression which might appreciably promoted the degradation of *GYS2* mRNA (Fig. 7l). All these data indicate that *GYS2* mRNA is the direct methylation substrate of METTL3 with an IGF2BP2-dependent manner in TSC.

Finally, we examined whether glycogen storage is linked to enhanced *GYS2* expression in TSC. As predicted, *METTL3* overexpression led to excessive glycogen storage (Fig. 7n), which were counteracted by *GYS2* knockdown (Fig. 7m–o). These experimental results indicate that GYS2 is a key target of METTL3 regulating glycogen storage in TSC.

### Targeted inhibition of *Mettl3* or *Gys2* suppresses the liver glycogen storage and lesions in vivo

**Fig. 8:**
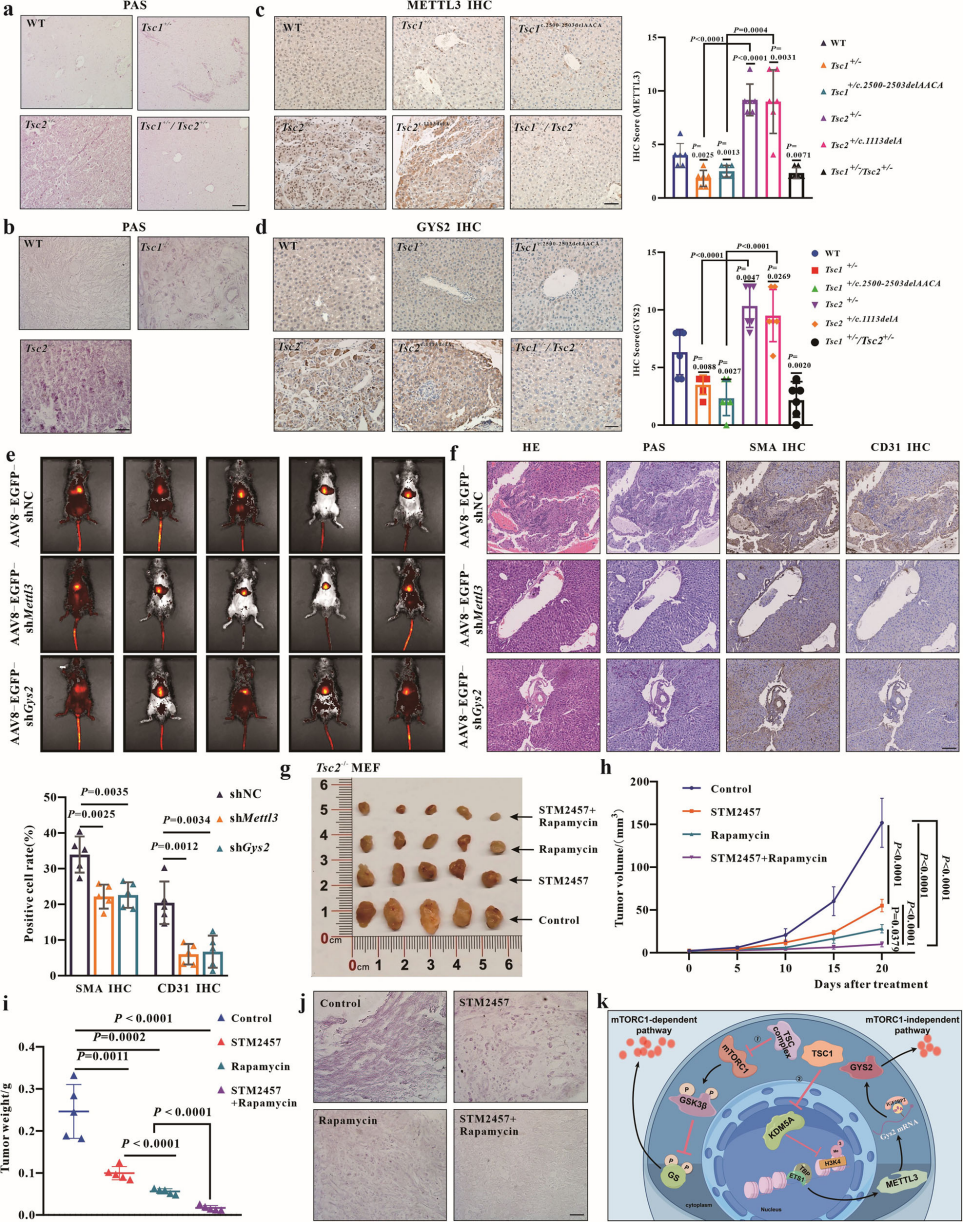
Targeted inhibition of METTL3 and GYS2 introduces the reduced glycogen level and liver lesions. **a** Representative images of PAS staining in liver tissues isolated from mice aged 12 to 13 months (WT, *Tsc1*^+/−^, *Tsc2*^+/−^ and *Tsc1*^+/−^/*Tsc2*^+/−^). *n* = 6 for each genotype; each group contains mice from three different litters. Bar, 100 μm in (**a**). **b** Representative images of PAS staining from tumor of nude mice (WT, *Tsc1*^−/−^, *Tsc2*^−/−^). *n* = 5 for each genotype. Bar, 50 μm in (**b**). **c** Representative images of METTL3 IHC staining in liver tissue isolated from mice aged 13 to 14 months and METTL3 IHC scores of all groups. *n* = 6 for each genotype; each group contains mice from three different litters. Bar, 50 μm in (**c**). **d** Representative images of GYS2 IHC staining in liver tissue isolated from mice aged 13 to 14 months and GYS2 IHC scores of all groups. *n* = 6 for each genotype; each group contains mice from three different litters. Bar, 50 μm in (**d**). **e** In vivo fluorescence imaging of all mice injected rAAV8, *n* = 5 for each group; each group contains mice from three different litters. **f** Representative images of HE staining, SMA IHC staining, CD31 IHC staining and PAS staining in liver tissues isolated from mice injected rAAV8 after 6 weeks. *n* = 5 for each group; each group contains mice from three different litters. Bar, 100 μm in (**f**). **g** Tumor sizes in each group at 20 days post drug treatment, *n* = 5 for each group. **h** The tumor volume in each group were monitored every 5 days starting from 1 day post drug treatment. *n* = 5 for each group. **i** The tumor weight in each group 20 days after drug treatment. **j** Representative images of PAS staining from tumor 20 days post drug treatment of nude mice. *n* = 5 for each group. Bar, 50 μm in **j**. **k** Schematic of the proposed mechanism, created by Figdraw. Data are shown as the mean ± SD, *n* = 5 ~ 6, two-way RM ANOVA and Tukey’s multiple comparisons test for (**h**), two-tailed unpaired Student’s *t*-test for others.

To assess the applicability of the TSC1-KDM5A-METTL3-IGF2BP2-GYS2 axis for TSC patients, *Tsc1*^+/−^, *Tsc2*^+/−^ and *Tsc1*^+/−^/*Tsc2*^+/−^ mice were bred (Fig. S7a–c), and tumorigenicity assays in nude mice were conducted for further validation. Glycogen levels in the livers of all knockout mice and tumors in nude mice were tested, and the results showed that glycogen levels were elevated with significantly greater increases in *Tsc2*^+/−^ mice and *Tsc2*^−/−^ tumors (Fig. 8a, b). However, primary MEFs isolated from day 12.5 embryos showed no changes in glycogen levels (Fig. S8a), possibly due to the slow glycogen storage progression in heterozygous mice. Liver protein expression assay revealed the decreased levels of both METTL3 and GYS2 in *Tsc1*-deficient mice, while increased levels in *Tsc2*-deficient mice consistent with our in vitro results. (Fig. 8c, d). To further confirm our findings, recombinant adeno-associated virus serotype 8 particles (rAAV8) were injected via the tail vein to specifically knockdown the liver expression of METTL3 or GYS2 in 10.5-month-old *Tsc2*^+/−^ mice. The in vivo fluorescence imaging and liver immunofluorescence reveal the liver targeting specificity of rAAV8 (Figs. 8e, and S8b, c). The knockdown efficiency of *Mettl3* or *Gys2* in mouse livers by rAAV8-EGFP-sh*Mettl3* or rAAV8-EGFP-sh*Gys2* injection was also confirmed by WB (Fig. S8d). Consistent with in vitro results, the mouse liver *Mettl3* or *Gys2* knockdown group exhibited the reduced glycogen accumulation, relieved liver lesions and decreased SMA and CD31 positive cell rates, indicating that *Mettl3* or *Gys2* knockdown could reduce the liver glycogen levels and relieve the liver lesions in vivo (Fig. 8f).

STM2457 is a small molecule that selectively inhibits METTL3, with potential therapeutic applications for cancer treatment [37, 38]. To further validate the role of METTL3 in TSC tumorigenesis in vivo, we performed a xenograft tumor formation assay using nude mice. Consistent with in vitro results, in vivo results indicated that STM2457 or rapamycin treatment significantly reduced tumor size and glycogen levels. Combination treatment with rapamycin and STM2457 led to the most significant reductions in tumor size and glycogen levels (Fig. 8g–j). These findings illustrate that targeting METTL3 and GYS2 is a promising combination therapy strategy with rapamycin for TSC patients with *TSC2* mutations.

## Discussions

Meikle et al. found that the loss of TSC1 in ventricular myocytes resulted in the increase of glycogen storage [39]. Pal et al. observed the enhanced glycogen storage in *Tsc2*^–/^^–^ MEFs [24]. However, no evidence clarifies the correlation of glycogen accumulation and disease progression in TSC. In cell and mouse models, we confirmed that the deletion of TSC1 and TSC2 led to the increased glycogen accumulation, with a more pronounced increase observed in TSC2 defect models. Additionally, we firstly revealed that glycogen accumulation may promote the progression of TSC-related liver lesions, which may be one important reason why patients with *TSC2* mutants exhibit more severe liver lesions. Our study provides crucial evidence linking glycogen accumulation to clinical disease manifestations.

GSK3β is one of the effector molecules downstream of mTORC1. Pal et al. has reported that the hyperactivation of mTORC1-GSK3β axis increase glycogen synthesis in *Tsc2*^*–/–*^ MEFs [24]. Our results further confirm that the increased mTORC1-GSK3β signaling enhances glycogen synthesis not only in *TSC2*-defective cells but also in *TSC1*-defective cells. Consistent with in vivo results, the results showed that the increased glycogen storage is much more pronounced in *Tsc2*^*–/–*^ cells than that in *Tsc1*^*–/–*^ cells.

METTL3 plays an important role in abnormal glucose metabolism. Shen et al. found that METTL3 stabilizes the expressions of HK2 and SLC2A1 (GLUT1) in colorectal cancer through an m^6^A-IGF2BP2/3-dependent mechanism, further activated the glycolysis pathway [40]. Gao et al. reported that METTL3 upregulated the m^6^A modification of NFAT5, which stabilizes NFAT5 mRNA and increased the expression of the gluconeogenesis-related genes GLUT1 and PGK1, resulting in enhanced aerobic glycolysis, proliferation, and tumor metastasis of intrahepatic cholangiocarcinoma [41]. In our study, METTL3 is firstly confirmed to be involved in abnormal glycogen storage in TSC.

If a functional complex is encoded by two genes, knocking out one gene will lead to an accumulation of its binding partner as uncomplexed form, which may cause unanticipated physiological or pathological consequences. Nevertheless, this paradigm remains largely unexplored in TSC pathogenesis. Our research confirmed that uncomplexed-TSC1 significantly upregulates the expression of METTL3, thereby driving m^6^A modification-dependent glycogen metabolism reprogramming in TSC. Redundant uncomplexed-TSC1 acts as a novel “gain-of-function” due to *TSC2* deficiency or *TSC1* overexpression.

Histone modifications modulate gene expression by changing the chromatin structure or recruiting other modifications[42], thereby involving numerous biological processes [43, 44]. We found that KDM5A-mediated H3K4me3 modification in the *METTL3* promoter facilitates its transcription. It’s reported that *Gys2*, the liver-specific glycogen synthase, is a substrate of METTL3 and plays a critical role in m^6^A-mediated glycogenesis [36]. In our study, our results are consistent with those previously reported in the literature. GYS2 is identified as the target of METTL3 in an IGF2BP2-dependent manner to promote glycogen storage. More importantly, targeted inhibition of the *Mettl3* or *Gys2* expression in liver effectively reduces the hepatic glycogen accumulation and liver injury in TSC.

However, this finding presents a thought-provoking contrast to research in the field of idiopathic pulmonary fibrosis (IDF). In IDF, METTL3 has been reported to increase m^6^A modification of *TSC1* mRNA, ultimately leading to a decrease in TSC1 protein expression [45]. We propose that the core of this seemingly contradictory phenomenon lies in the state of the TSC1 protein. In TSC, it was the free, uncomplexed-TSC1 monomer that exerted a positive regulatory effect on METTL3. This uncomplexed-TSC1 act as a novel “gain-of-function”. In contrast, in the study of IDF, TSC1 protein produced from METTL3-downregulated mRNA is primarily involved in forming the TSC1-TSC2 complex. Additionally, the differences in disease contexts may lead to distinct underlying mechanisms.

In summary, our findings revealed that the hyperactivation of mTORC1-GSK3β signaling enhances glycogen synthesis, while uncomplexed-TSC1 through the TSC1-KDM5A-METTL3-IGF2BP2-GYS2 axis employs mTORC1-independent pathways to exacerbate glycogen storage in TSC with *TSC2* defects but slightly alleviates glycogen storage in TSC with *TSC1* defects (Fig. 8k). This may be one reason why patients with *TSC2* mutations have more severe liver lesions. The combined treatment of mTORC1 and *METTL3* or *GYS2* inhibitors may be more effectively suppress liver lesions and glycogen accumulation in TSC with *TSC2* defects, indicating the potential for precision medicine in TSC. Our finding will provide a potential therapeutic strategy for rare disease caused by a defected complex.

## Materials and methods

### Animal models

*Tsc1* heterozygous knockout mice (*Tsc1*^*+/−*^), *Tsc2* heterozygous knockout mice (*Tsc2*^*+/−*^), *Tsc1* heterozygous mutant mice (*Tsc1*^c.2500-2503delAACA^), *Tsc2* heterozygous mutant mice (*Tsc2*^c.1113delA^) and BALB/c nude mice (18–20 g, 6 weeks) were purchased from GemPharmatech (Nanjing, China). *Tsc1* and *Tsc2* double heterozygous knockout (*Tsc1/2*^*+/−*^) mice were obtained by cross breeding *Tsc1*^*+/−*^ with *Tsc2*^*+/−*^. The PCR primers used for genotyping of each mouse strain were listed in Table S1. All mice were on the C57BL/6 genetic background. The Committee of Scientific Research in Shanxi University (CSRSX) reviewed and approved all animal experiments. The approval number is SXULL2020032. All animal experiments were carried out by following a protocol approved by the Institutional Research Ethics Committee of Shanxi University.

### Cell culture

The immortalized HepG2 cell line was preserved by our lab. Immortalized WT MEFs, *Tsc*1^−/−^ MEFs, and *Tsc*2^−/−^ MEFs were gifts from Prof. Hongbing Zhang (Peking Union Medical College, China). *Tsc1/*2^−/−^ MEFs was generated by transforming *Tsc*2^−/−^ MEFs with the lentiviral CRISPR-Cas9 system to target *TSC1*. The sgRNA sequence was as follows: 5′-GCCTATGCTTGTCAACACGTTGG-3′. Primary mouse embryonic fibroblasts (WT MEFs, *Tsc*1^+/−^ MEFs and *Tsc*2^+/−^ MEFs) were isolated from embryos at embryonic day 12.5. All cell lines were cultured and maintained in Dulbecco’s modified Eagle’s medium (DMEM) with 10% fetal bovine serum (FBS) and 1% penicillin–streptomycinat at 37 °C and 5% CO_2_.

### RNA interference and plasmid transfection

Knockdown of *TSC1*, *TSC2*, *WTAP*, *METTL3*, *KDM5A*, *GYS2* and *IGF2BP2* in HepG2 or MEFs was achieved by transfection with siRNA (GenePharma, Shanghai, China) using Lipofectamine 3000 (Invitrogen) according to the manufacturer’s instructions. The siRNA sequences are listed in Table S2. After verifying the efficient silence of the target genes, the cells were used in the following experiments. The pcDNA3.1-myc-*TSC1* and pcDNA3.1-Flag-*TSC2* plasmids were preserved by our lab; pcDNA3.1-*METTL3*-WT-3*HA was purchased from PPL (Jiangsu, China); pcDNA3.1-*METTL3*-mut-3*HA were constructed in our Lab; pcDNA3.1-*KDM5A*-Flag purchased from Cyagen (Suzhou, China) for overexpression experiments. PEI transfection reagents supplied by YEASEN (Shanghai, China) were used for transfection.

### Western blotting

Cells for protein analysis were collected, and total proteins were extracted using RIPA buffer (BOSTER, Wuhan, China) with phosphatase and protease inhibitors. The BCA Protein Assay kit (Thermo Fisher Scientific, Massachusetts) was used to determine the protein concentrations. The proteins were separated by SDS-polyacrylamide electrophoresis (SDS-PAGE) and then transferred onto PVDF membranes. Blocking of the membranes was achieved by incubation in 5% defatted milk for 2 h. The primary antibody was then applied overnight at 4 °C, following by three washes with TBST, incubation with the secondary antibody at RT for 1 h, followed by three additional washes. The information for primary antibodies is shown in Table S3. Finally, the bands were visualized using the enhanced chemiluminescence kit (Millipore, Massachusetts).

### qRT-PCR

Total RNA was extracted using TRIzol method, and cDNA was synthesized using PrimeScript™ RT reagent Kit with gDNA Eraser (Takara, RR047A) according to the manufacturer’s instructions. The Takara PrimeScript™ One Step RT-PCR Kit was employed for the RT-PCR assay (Takara, RR055A). PCR primer sequences are shown in Table S4.

### Magnetic Resonance Imaging (MRI)

MRI was conducted by Shanxi Medical University and performed as described in this article [46].

### Hematoxylin and Eosin (HE) staining

HE staining was conducted by Department of Pathology, The Second Hospital of Shanxi Medical University (Shanxi, China). The pre-prepared paraffin sections were first de-paraffinized with xylene (I) and (II) for 5 min each. This was followed by treatment with a gradient of ethanol, 100% ethanol: 3 min, 95%: ethanol 3 min, 90% ethanol: 3 min, 80% ethanol: 3 min, 70% ethanol: 3 min, concluding with rinsing in ddH_2_O for 3 min. The de-paraffinized tissue sections were then stained with hematoxylin staining solution for 20 min and washed with tap water for 10 min. Subsequently, a differentiation solution was applied for 30–60 s, and the tissue samples were soaked in water for 10 min. The samples were stained with eosin staining solution for 30–60 s and rinsed under running water for 5 min. Finally, they underwent ethanol gradient dehydration, were cleared with xylene, and sealed with neutral gum.

### Periodic Acid–Schiff (PAS) staining

Glycogen Periodic Acid Schiff (PAS) Stain Kit (For Cells) (Solarbio, G1360) was used to detect intracellular glycogen. The cells were fixed with PAS Fixative for 15 min, after washing off the fixative with distilled water and then air drying, then cells were treated with Oxidant at room temperature for 20 min. Twice washing with distilled water, each time for 2 min. Samples were incubated in Schiff staining solution at room temperature in the dark for 20 min, followed by rinsing with running water for 5 min. Finally, samples were incubated in Mayer Hematoxylin Staining Solution for 1 min, washed and dried, and visualized under the microscope.

### Glycogen content assay

The Glycogen Content Assay Kit (Solarbio, BC0345) was used to quantify glycogen. In brief, cells (5 × 10^6^) were collected and centrifuged at 3000 × *g* for 5 min, after removal of the supernatant, extract (provided with the kit) was added. Cells were fragmented by ultrasound in an ice bath, and then incubated in boiling water bath for 10 min. After cooling, resuspended in 5 ml of distilled water, the samples were centrifuged at 8000 × *g* for 10 min at 25 °C. The supernatant was mixed with reagent 1 and reagent 2 (provided with the kit), and then boiled for 10 min, the OD value of each cooled 200 µl of sample was measured in 96-well plate in triplicate at 620 nm.

### RNA m^6^A quantification

TRIzol (Thermo Fisher, Massachusetts) was used for RNA extraction from cells, and NanoDrop3000 was used to quantify and qualify the RNA. RNA m^6^A was measured using the EpiQuik m^6^A RNA Methylation Quantification Kit (Colorimetric) (P-9005, Epigentek, USA). 300 ng of total RNA was added to each well, along with the manufacturer’s capture antibody solution and detection antibody solution. Absorbance at 450 nm was measured using a microplate reader to calculate the m^6^A levels.

### M^6^A dot blot

The RNA was extracted and prepared as described above. For RNA m^6^A dot blot assay, RNA was denatured at 95 °C for 5 min, then cross-linked by irradiating with UV light at 254 nm. The m^6^A antibody was incubated overnight with the nylon membrane. The membrane was then exposed to a visualizer (GE Healthcare, Pittsburgh). RNA on the membrane was verified using methylene blue staining.

### mRNA stability

Total RNA was extracted from cells at 0 h, 2 h, 4 h, 8 h,10 h post-treatment of Actinomycin D (Act-D, 5 μg/mL) (MCE, China, Shanghai), mRNA expressions were quantified using qRT‒PCR.

### Protein stability

Protein stability of targets in MEFs was assessed by incubating with cycloheximide (CHX,100 μg/ml) (MCE, China, Shanghai) for the indicated times. The expression of METTL3 was measured using Western blot analysis.

### RNA-seq

RNA-seq was performed by Shanghai Bioprofile Technology Co., Ltd (Shanghai, China). In brief, total RNA was extracted using Trizol Reagent. Purified RNA was quantified at OD260 nm using an ND-1000 spectrophotometer (NanoDrop Technologies, Inc. Wilmington, DE, USA) and qualified using a Bioanalyzer 2100 (Agilent Technologies, Inc.) with RNA 6000 LabChip kit (Agilent Technologies, Inc.). RNA-Seq libraries were constructed using Agilent’s SureSelect Strand-Specific RNA Library Preparation Kit, followed by AMPure XP Beads (Beckman Coulter, Brea, CA, USA) size selection, and the sequence was directly determined using Illumina’s sequencing-by-synthesis technology.

### Immunofluorescence staining

First, 4% paraformaldehyde was used to fix cells on slides in 24-well plates, Then, Triton X-100 (0.5%) was used to permeabilize cells for 10 min, followed by three washes with PBS, blocked with 5% bovine serum albumin (BSA) in PBS for 2 h, rewashed three times with PBS, and then incubated with primary antibodies overnight at 4 °C. After washing three times, the slides were then incubated with secondary antibodies for 1 h at room temperature. After washing three times, then the slides were incubated with DAPI (Solarbio) for 5 min at room temperature, and images were captured using a laser scanning confocal microscope (Zeiss, Oberkochen, Germany). The primary antibodies information shows in Table S3.

### Immunohistochemistry (IHC)

All slides were placed in a 60 °C incubator for 20 min, deparaffinized in xylene and rehydrated in gradient ethanol. The slides were incubated with 3% hydrogen peroxide for 10 min, followed by antigen retrieval using 0.01 M citrate buffer (pH 6.0) for 30 min. After blocking with 5% BSA, slides were incubated overnight at 4 °C with relevant primary antibody. Subsequently, the secondary biotin-conjugated antibody was applied for 1 h at room temperature. The IHC staining was visualized using a diaminobenzidine reaction and counterstained with hematoxylin.

### Sucrose density gradient centrifugation

Sucrose density gradient centrifugation was performed as described previously [47]. Sucrose dissolved in lysis buffer was used to create gradient layers of 25% (0.5 ml), 27.5% (0.5 ml), 30% (0.5 ml), 32.5% (0.5 ml), 35% (0.5 ml), 37.5% (0.5 ml), 40% (0.5 ml) and 45% (0.5 ml) in 6 ml ultracentrifuge tubes. Then, 0.5 ml protein lysate was layered on top of the sucrose solution and centrifuged over the gradients at 4 °C at 185,000 × *g* for 18 h. Nine fractions of 0.5 ml volume were collected and analyzed by Western blotting.

### ChIP assay

ChIP assays were performed using the ChIP Assay Kit (P2078, Beyotime). Briefly, cells were cross-linked with 1% formaldehyde, lysed, and sonicated on ice to generate DNA fragments with an average length of 200–1000 bp. Precleared DNA from each sample was saved as an input fraction. This precleared DNA was then used for immunoprecipitation with 5 μg of ChIP-grade antibody. And IgG was included as a nonspecific control. DNA was eluted and purified, followed by RT-qPCR using specific primers. The PCR primer sequences are shown in Table S5.

### rAAV8-mediated shRNA liver-targeted delivery experiment

The rAAV8 particles were produced by Applied Biological Materials Inc (Vancouver, Canada). The stock of virus was diluted in saline to a concentration of 5 × 10^11^/0.2 mL. Subsequently, each mouse received a tail vein injection of 0.2 mL of the viral dilution. Three weeks later, in vivo fluorescence imaging was performed to assess the efficacy of viral delivery to the liver.

### In vivo subcutaneous implantation models

For the subcutaneous implantation model, WT MEFs, *Tsc*1^−/−^ MEFs and *Tsc*2^−/−^ MEFs were injected subcutaneously into BALB/c nude mice. For models, 1 × 10^7^ MEFs in 0.2 ml solvent control were injected into the backs of nude mice. For tumorigenicity assays, five days later, the tumor cell-inoculated mice were randomly divided into four treatment groups with five mice per group: the solvent control group, STM2457 group, rapamycin group, and rapamycin combined with STM2457 group (the dose of STM2457 was 30 mg/kg, and the rapamycin dose was 1 mg/kg). STM2457 and rapamycin by intraperitoneal injection every day. Tumors were measured with a caliper every 5 days and the tumor volume was calculated using the formula *V* = 0.52(width^2^× length). After 20 days, the mice were killed, and the tumors were resected and weighed.

### Statistical analysis

All data were analyzed using Prism 5.0 (GraphPad, San Diego, CA, USA) and expressed as mean ± SD from at least three independent experiments. For statistical analysis, a two-tailed unpaired Student’s *t*-test was performed between two groups, a two-way repeated-measures ANOVA was used to compare the effects of [Factor A] and [Factor B] across multiple time points. And a *p*-value of <0.05 was considered statistically significant.

## Supplementary information

Supplementary Information

Supplementary Figure

Full and uncropped western blots

## Data Availability

The datasets generated during the current study are available from the corresponding author upon reasonable request.
