# Supplementary material for: Uncomplexed-TSC1 deploys novel mTORC1-independent pathway to exacerbate the liver glycogen storage in TSC
Source: Cell Death Dis. 2025 Nov 14;16(1):829. doi: 10.1038/s41419-025-08161-3 (PMC12618697; doi:10.1038/s41419-025-08161-3)
Supplement: Supplementary file 1 — Supplementary Information [file 41419_2025_8161_MOESM1_ESM.docx]

**Supplementary Table 1** Primers for genotyping

| **Primer Name** | **Primer sequence (5'—3')** | |
| --- | --- | --- |
|  | **Forward** | **Reverse** |
| ***Tsc1*^+/-^** | TGTCACTGAGCAGAAATGGCTACG | CTGCAATCAAATGCCTAGAGCAC |
| ***Tsc2*^+/-^** | GAGGACTGGGACTAAAGGTGTGTTC | AAAGGAAGTGTGTCACCGCG |
| ***Tsc1*^+/c.2500-2503 delAACA^** | GTGGCTTTCCATAGGTATTCAGTG | ACTGACATTCATGTAGCCTGGAC |
| ***Tsc2*^+/c.1113 delA^** | TCCTCTATGAGGCATAACACTGCC | TCACTTGGTCTGCCTTGGACAA |

**Supplementary Table 2** siRNA sequences

| **siRNA** | **siRNA sequence (5'—3')** | **species** |
| --- | --- | --- |
| **si-*TSC1*** | CCAAAUCUCAGCCCGCUUUTT | Human |
| **si-*TSC2*** | CAAUGAGUCACAGUCCUUUGATT | Human |
| **si-*Mettl3*-1** | GCACACUGAUGAAUCUUUATT | Mouse |
| **si-*Mettl3*-2** | CAGUCAUAAACCAGAUGAATT | Mouse |
| **si-*Wtap*-1** | GGAACAGACUAAAGACAAACUTT | Mouse |
| **si-*Wtap*-2** | GACCCAGCAAUCAACUUGUTT | Mouse |
| **si-*METTL3*-1** | GCAAGUAUGUUCACUAUGATT | Human |
| **si-*METTL3*-2** | CAAGGAACAAUCCAUUGUUTT | Human |
| **si-*GYS2*-1** | GUGGGAUGUUGCACAUUCUTT | Human |
| **si-*GYS2*-2** | CACCACGGUUUCUGAAAUATT | Human |
| **si-*KDM5A*-1** | AAGCGAUUCUGGGGUUUCUGUGUTT | Human |
| **si-*KDM5A*-2** | CUCCAUUUGCCUGUGAAGUAAAATT | Human |
| **si-*WTAP*-1** | GCUUUGGAGGGCAAGUACATT | Human |
| **si-*WTAP*-2** | GGUUCGAUUGAGUGAAACATT | Human |
| **si-*IGF2BP2*** | CGGATCTTTGGGAAACTGAAA | Human |

**Supplementary Table 3** Antibodies

| **Product name** | **Manufacturer** | **Catalog No.** |
| --- | --- | --- |
| **TSC1** | CST | #6935 |
| **TSC2** | CST | #4308 |
| **p-P70S6(Thr389）** | CST | #9205 |
| **T-P70S6** | CST | #34475 |
| **β-actin** | Proteintech | 66009-1-Ig |
| **T-GSK3β** | Proteintech | 22104-1-AP |
| **p-GSK3β(S9)** | Bioss | bs-2066R |
| **m6A** | HUABIO | #HA721152 |
| **METTL3** | Proteintech | 15073-1-AP |
| **WTAP** | Proteintech | 10200-1-AP |
| **METTL14** | Proteintech | 26158-1-AP |
| **FTO** | Proteintech | 27226-1-AP |
| **ALKBH5** | Proteintech | 67811-1-Ig |
| **TBP** | Abclonal | 22006-1-AP |
| **NRF1** | Abclonal | A3252 |
| **ETS1** | Proteintech | 66598-1-Ig |
| **KDM5A** | zen-bioscience | R382348 |
| **GYS2** | CST | #71217 |
| **SMA** | zen-bioscience | R23450 |
| **CD31** | zen-bioscience | R347526 |
| **HMB45** | Immunoway | ABT255 |
| **H3K4me3** | Abcam | ab213224 |
| **IGF2BP2** | zen-bioscience | R389232 |

**Supplementary Table 4** Primers for RT-qPCR

| **Primer Name** | **Primer sequence (5'—3')** | |
| --- | --- | --- |
|  | **Forward** | **Reverse** |
| **(h)*METTL3*** | TTTTCCGGTTAGCCTTCGGG | GATAGAGCTCCACGTGTCCG |
| **(h)*GYS2*** | TGAAGTTGCTTGGGAAGTGAC | TGAAGTTGCTTGGGAAGTGAC |
| **(h)*G6PC*** | CGAGGCGCTACAGAACCAG | CACTCGGTGATGAGGCTGAT |
| **(h)*GSK3β*** | GGAGGAACTCACCGACCTTTG | CGTCCGTAACGCTTCCCAC |
| **(h)*KDM5A*** | GTCTAAAGTGGGTAGTCGCTTG | GTTTGGGTATCAGTGCTGAGAA |
| **(h)*ZRANB3*** | TTAACCGCAGATGCAAAGACT | AGAATGGCTCGTCTGGCTTTC |
| **(h)*SOX9*** | AGCGAACGCACATCAAGAC | CTGTAGGCGATCTGTTGGGG |
| **(h)*CHD7*** | TGATGAGTCTTTTTGGCGAGG | CTGGATTTTCCGGGTAACCAC |
| **(h)*TP63*** | GGACCAGCAGATTCAGAACGG | AGGACACGTCGAAACTGTGC |
| **(h)*GCGR*** | GTGGAAGCTCTACGGTGACC | AGCAGGAATACTTGTCGAAGGT |
| **(h)*IGF2BP2*** | GTCCTACTCAAGTCCGGCTAC | CATATTCAGCCAACAGCCCAT |
| **(h)*ACTIN*** | TTGCTGACAGGATGCAGAAG | ACTCCTGCTTGCTGATCCACAT |
| **(m)*Actin*** | GAGACCTTCAACACCCCAGC | ATGTCACGCACGATTTCCC |
| **(m)*Mettl3*** | CTGGGCACTTGGATTTAAGGAA | TGAGAGGTGGTGTAGCAACTT |
| **(m)*Tbp*** | CTTCCTGCCACAATGTCACAG | CCTTTCTCATGCTTGCTTCTCTG |
| **(m)*Nrf1*** | GCTTAGCCCTCGATGAAGACT | GCTTAGCCCTCGATGAAGACT |
| **(m)*Ets1*** | GACGTGGGTTTCTGTCCACT | GATCTCAAGCCGACTCTCACC |

**Supplementary Table 5** Primers for ChIP-qPCR

| **Primer Name** | **Primer sequence (5'—3')** | |
| --- | --- | --- |
|  | **Forward** | **Reverse** |
| **Chip-*METTL3*** | **ACACAACGCAACCAGGACCT** | **TTTGTGTTGACGAGGCGTGG** |
