# Supplementary material for: Uncomplexed-TSC1 deploys novel mTORC1-independent pathway to exacerbate the liver glycogen storage in TSC
Source: Cell Death Dis. 2025 Nov 14;16(1):829. doi: 10.1038/s41419-025-08161-3 (PMC12618697; doi:10.1038/s41419-025-08161-3)
Supplement: Supplementary file 2 — Supplementary Figure [file 41419_2025_8161_MOESM2_ESM.docx]

Fig.S1


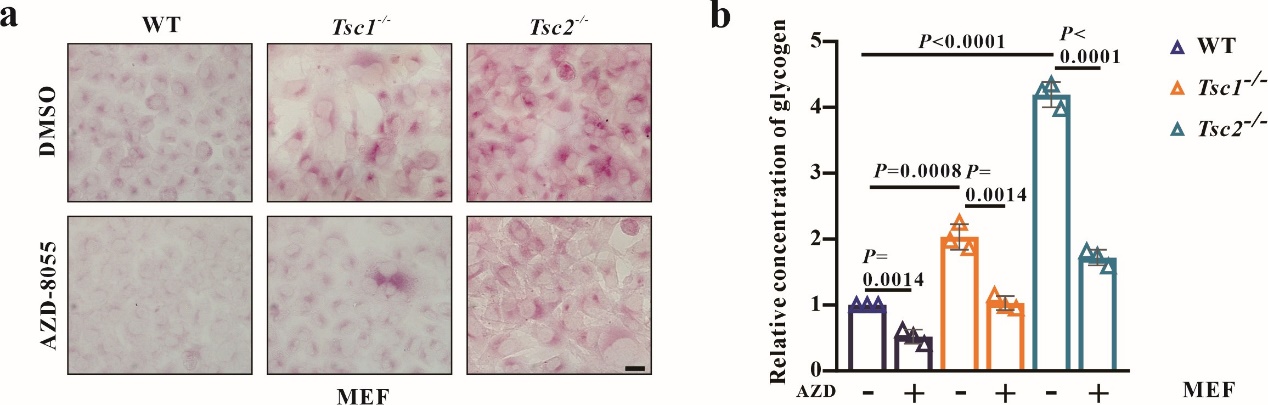


(**a and b)** Analysis of glycogen levels in MEFs (WT, *Tsc1*^−/−^, *Tsc2*^−/−^) at 24 h post treatment of 1μM AZD-8055 or control (DMSO) using PAS staining and glycogen assay kit. Bar, 20 μm in **a**.

Fig. S2


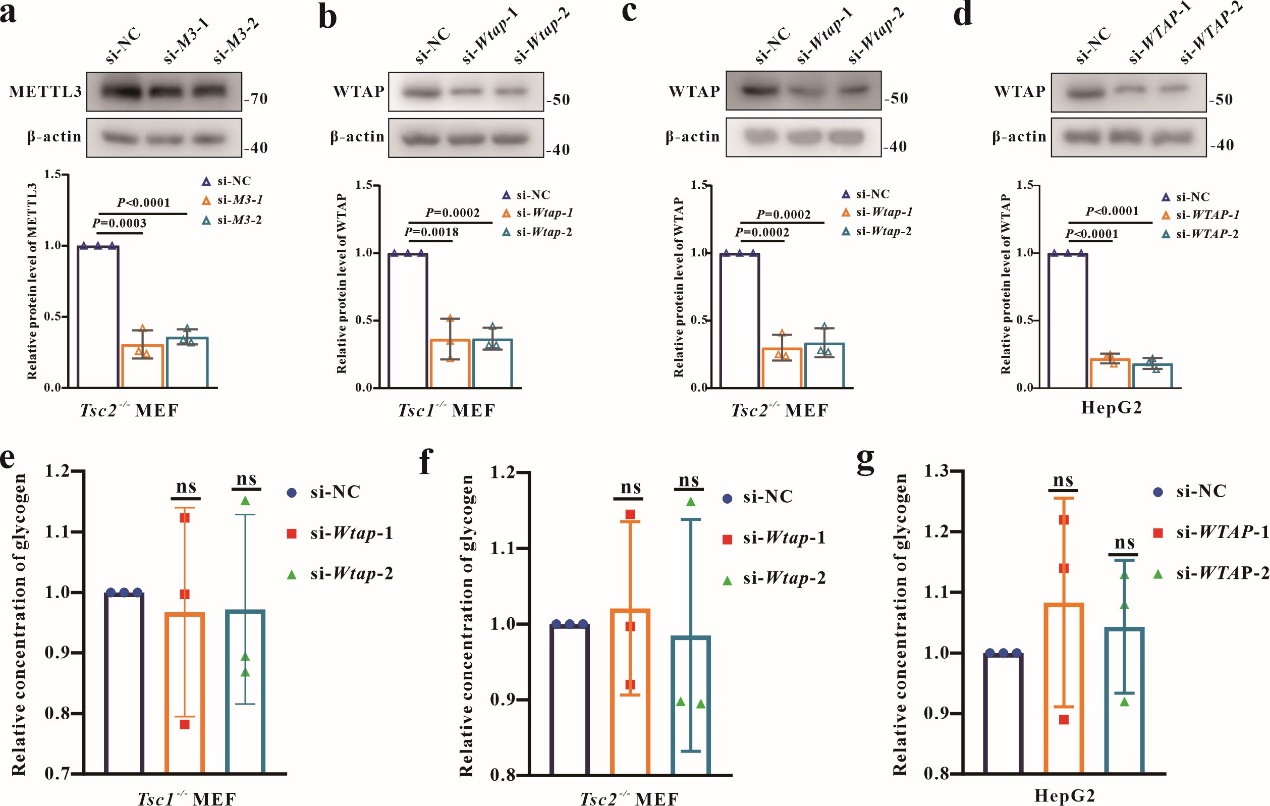


(**a**)WB quantification of knockdown efficiency of *Mettl3* in *Tsc2*^−/−^ MEFs (si-*M3*-1, si-*M3*-2 targeting mouse *Mettl3)*. (**b, c and d**) Western blot (WB) quantification of the knockdown efficiency of *WTAP* in *Tsc1*^−/−^ MEFs, *Tsc2*^−/−^ MEFs and HepG2 cells (si-*Wtap*-1, si-*Wtap*-2 targeting mouse *Wtap*; si-*WTAP*-1, si-*WTAP*-2 targeting human *WTAP*). (**e, f and g**) Detection of glycogen levels in *Tsc1*^−/−^ MEFs (si-NC, si-*Wtap*-1, si-*Wtap*-2), *Tsc2*^−/−^ MEFs (si-NC, si-*Wtap*-1, si-*Wtap*-2) and HepG2 cells (si-NC si-*WTAP*-1, si-*WTAP*-2).

Fig.S3


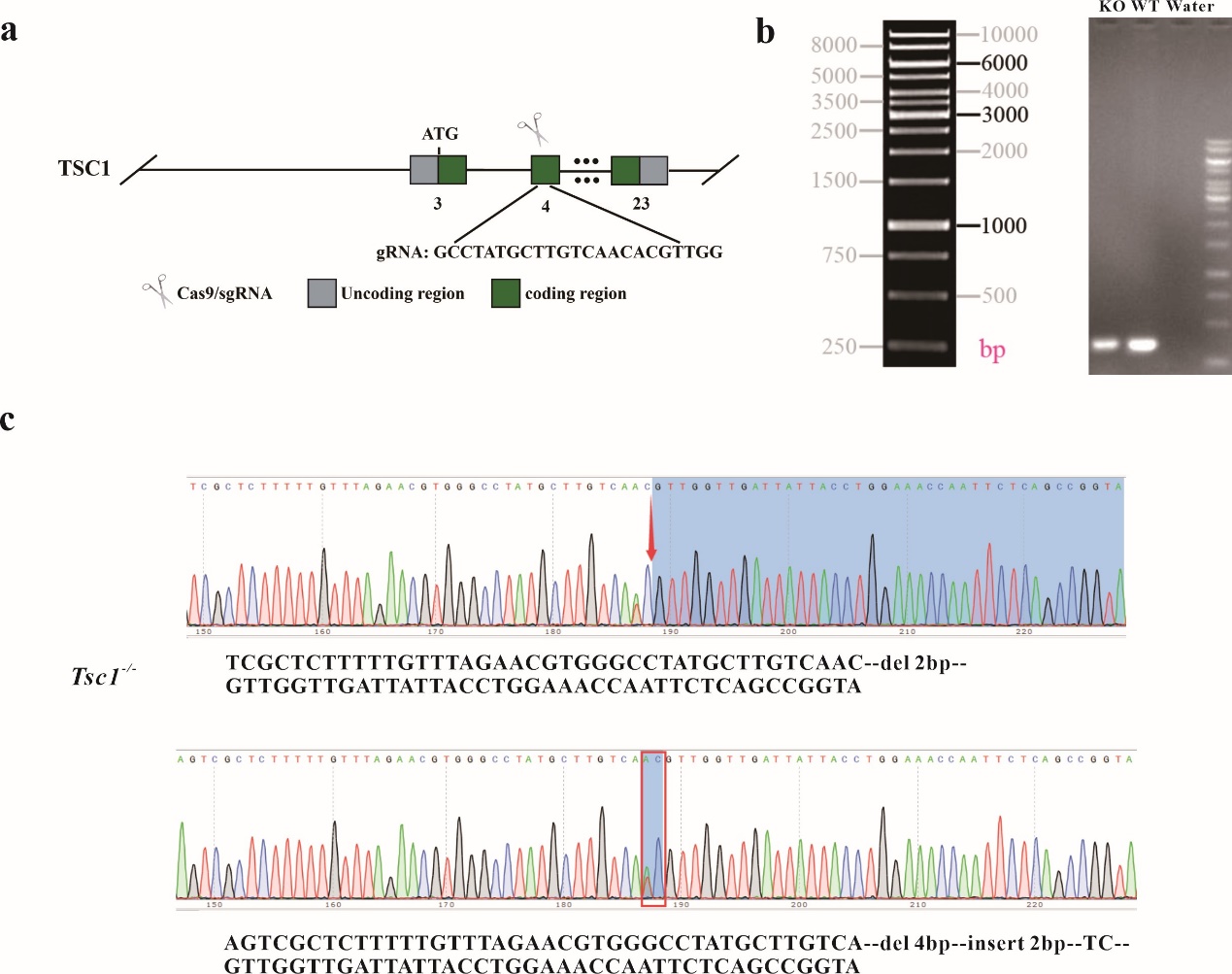


**(a)** Schematic diagram illustrating the knockout of *Tsc1* in *Tsc2^-/^* MEFs using Cas9/sgRNA, created with Figdraw. **(b)** PCR amplification of the *Tsc1* DNA fragment in cells with *Tsc1/2^-/-^* and *Tsc2^-/-^* genotype. (**c**) Confirmation of *Tsc1* knockout in *Tsc2^-/-^* cells using Sanger sequencing.

Fig.S4

**
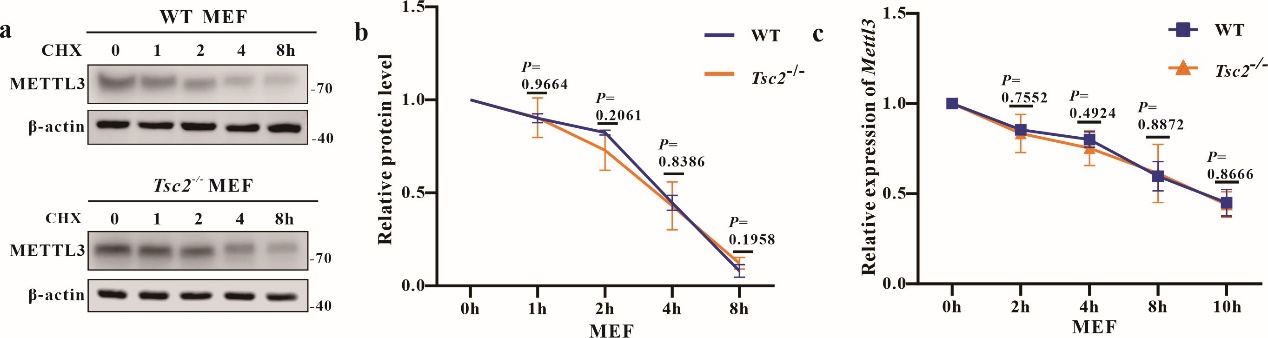
(a and b)** WB analysis and quantification of METTL3 in WT MEFs and *Tsc2*^-/-^MEFs at 0, 1 h, 2 h, 4 h and 8 h post-CHX treatment. **(c)** Quantification of METTL3 mRNA in WT MEFs, *Tsc2*^-/-^MEFs using qRT-PCR at 0, 2 h, 4 h, 8 h and 10 h post-Act-D treatment.

Fig.S5

**
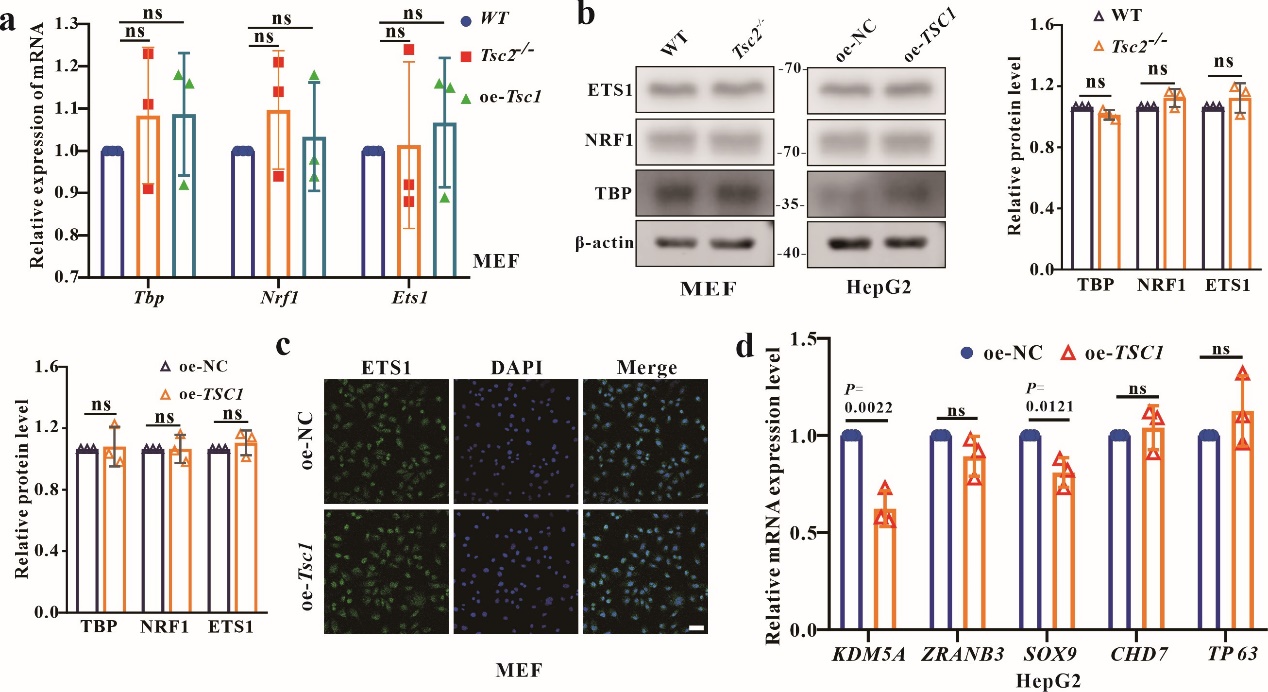
(a)** Analysis of *Tbp, Nrf1, Ets1* mRNA expression in WT MEFs, *Tsc2*^-/-^MEFs and oe-*Tsc1* MEFs using qRT-PCR*.* **(b)** Analysis of TBP, NRF1, ETS1 protein in WT MEFs, *Tsc2*^-/-^MEF, oe-NC and oe-*TSC1* HepG2 using WB. **(c)** Immunofluorescent visualization of ETS1 localization in MEFs under confocal microscope, Bar, 50 μm in **c**. **(d)** Analysis of *KDM5A, ZRANB3, SOX9, CHD7, TP63 (Trp63* in mouse) mRNA expression in oe-NC and oe-*TSC1* HepG2 using qRT-PCR.

Fig.S6


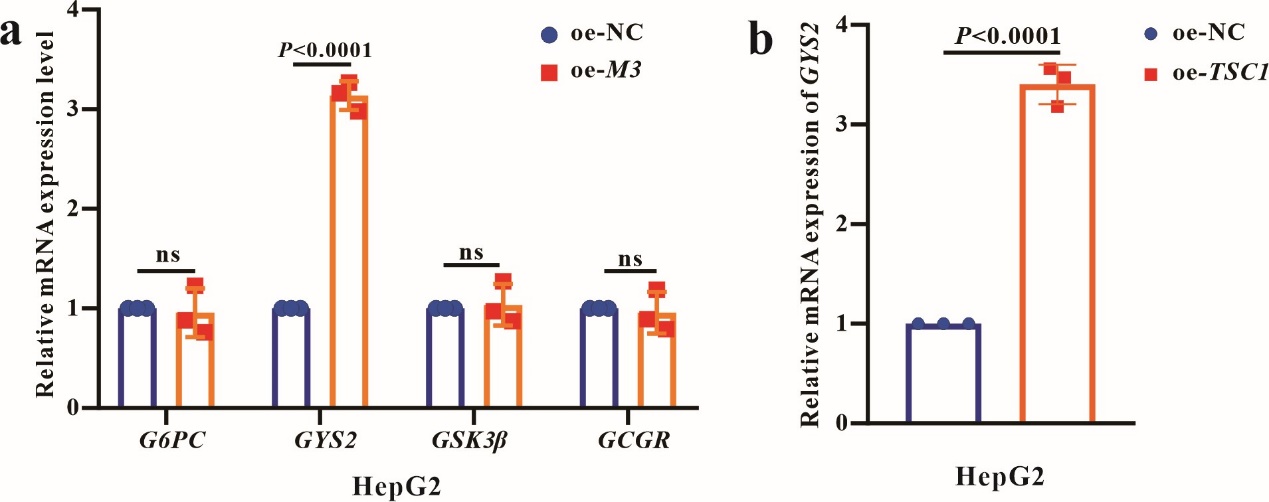


**(a)** Analysis of *G6PC*, *GYS2*, *GSK3β*, *GCGR* mRNA expression in oe-NC and oe-*METTL3* HepG2 using qRT-PCR. **(b)** Analysis of *GYS2* mRNA expression in oe-NC and oe-*TSC1* HepG2 using qRT-PCR.

Fig.S7


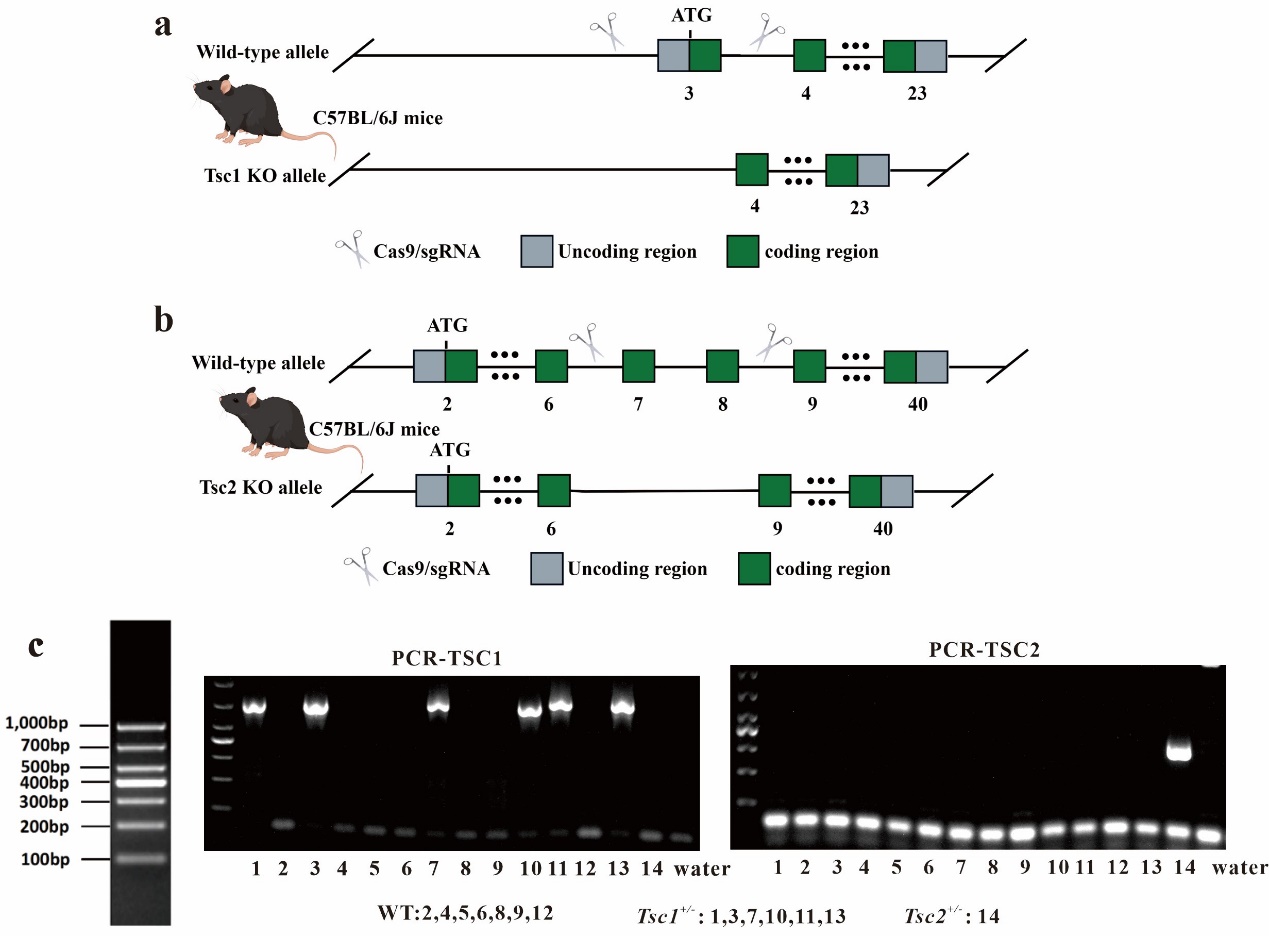


**(a)** Schematic diagram illustrating the generation of *Tsc1*^+/−^ mice using the CRISPR/Cas9 system, created with Figdraw. **(b)** Schematic diagram illustrating the generation of *Tsc2*^+/−^ mice using the CRISPR/Cas9 system, created with Figdraw. **(c)** Identification of mouse genotypes using PCR.

Fig.S8


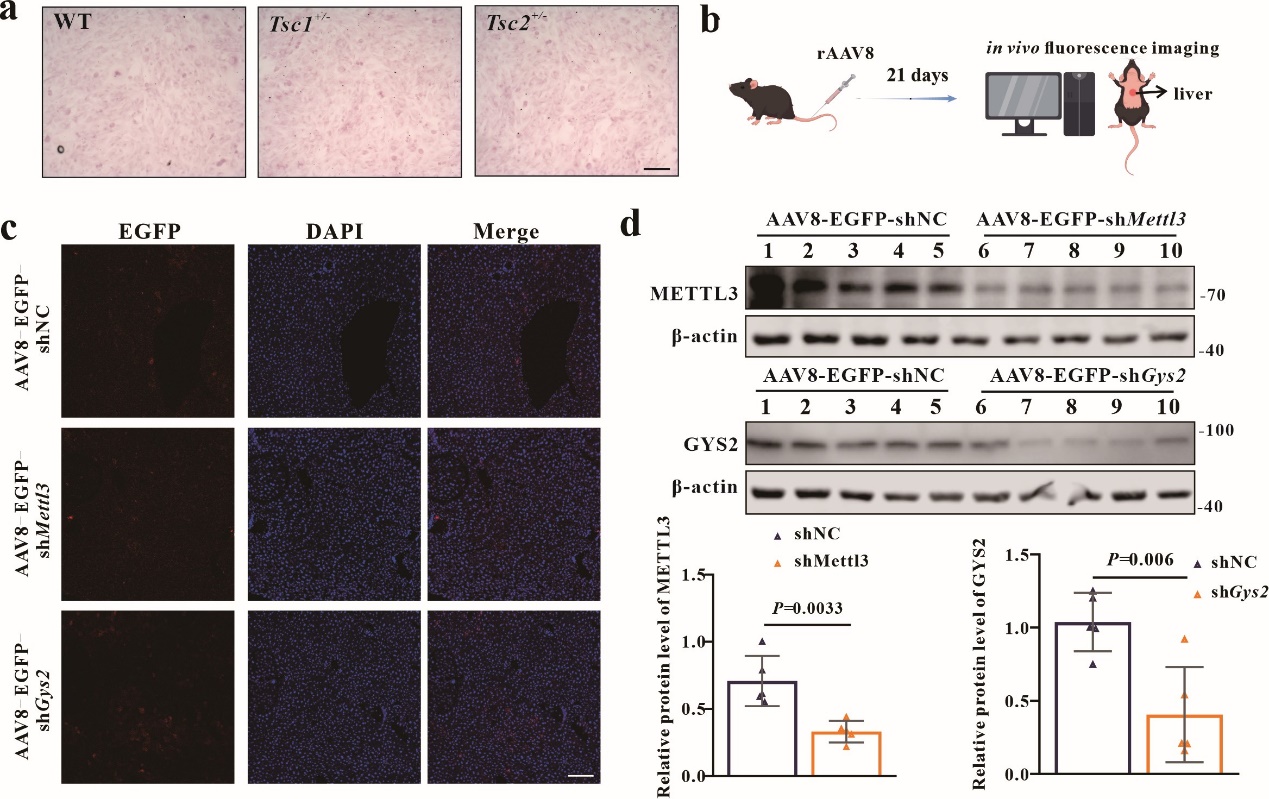


(**a**) Representative images of PAS staining in primary MEFs (WT, *Tsc*1^+/−^, *Tsc2*^+/−^). n = 3 for each genotype; each group contains mice from two different litters. Bar, 100 μm in **a**. (**b**) Schematic representation of the *in vivo* study, created with Figdraw. (**c**) *In vivo* fluorescence imaging to detect rAAV8 in mouse liver tissues *in vivo.* Bar, 100 μm in **c**. (**d**) Analysis Knockdown efficiency of AAV8-EGFP-sh*Mettl3* and AAV8-EGFP-sh*Gys2* in liver.
