# Supplementary material for: Uncomplexed-TSC1 deploys novel mTORC1-independent pathway to exacerbate the liver glycogen storage in TSC
Source: Cell Death Dis. 2025 Nov 14;16(1):829. doi: 10.1038/s41419-025-08161-3 (PMC12618697; doi:10.1038/s41419-025-08161-3)

Fig2a-MEF

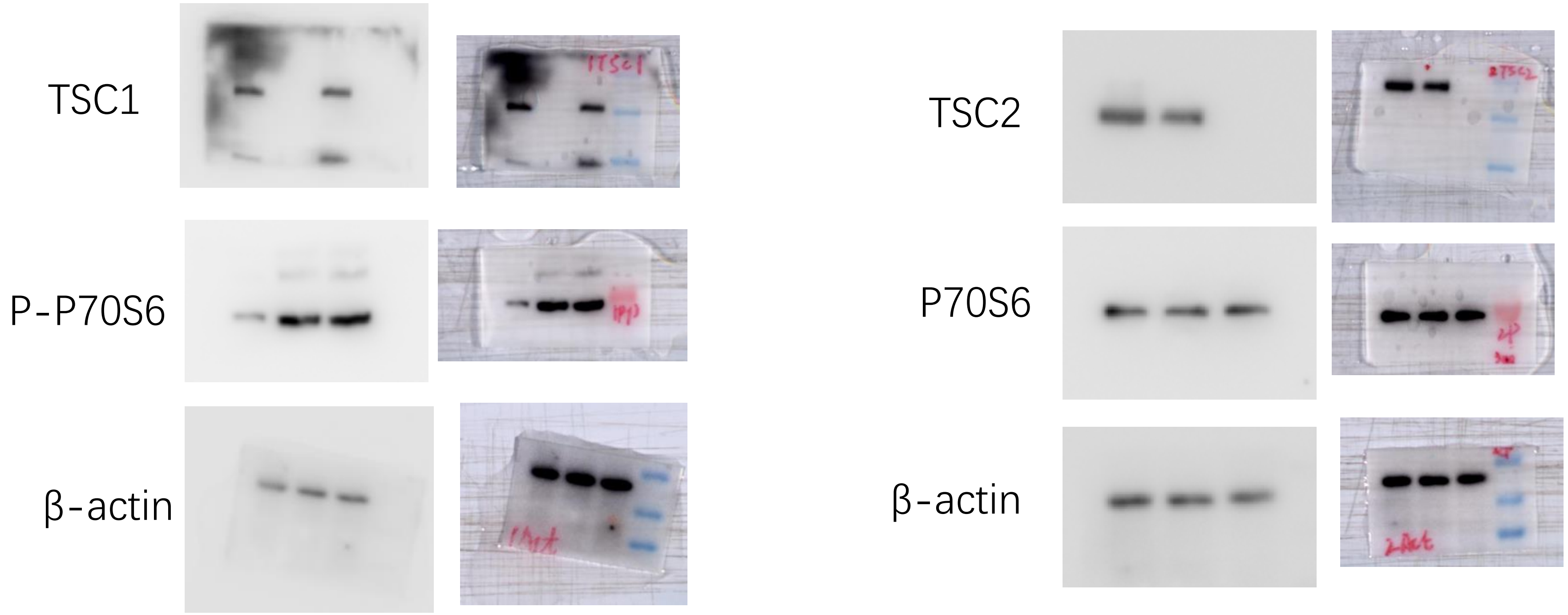

Fig2a-HepG2

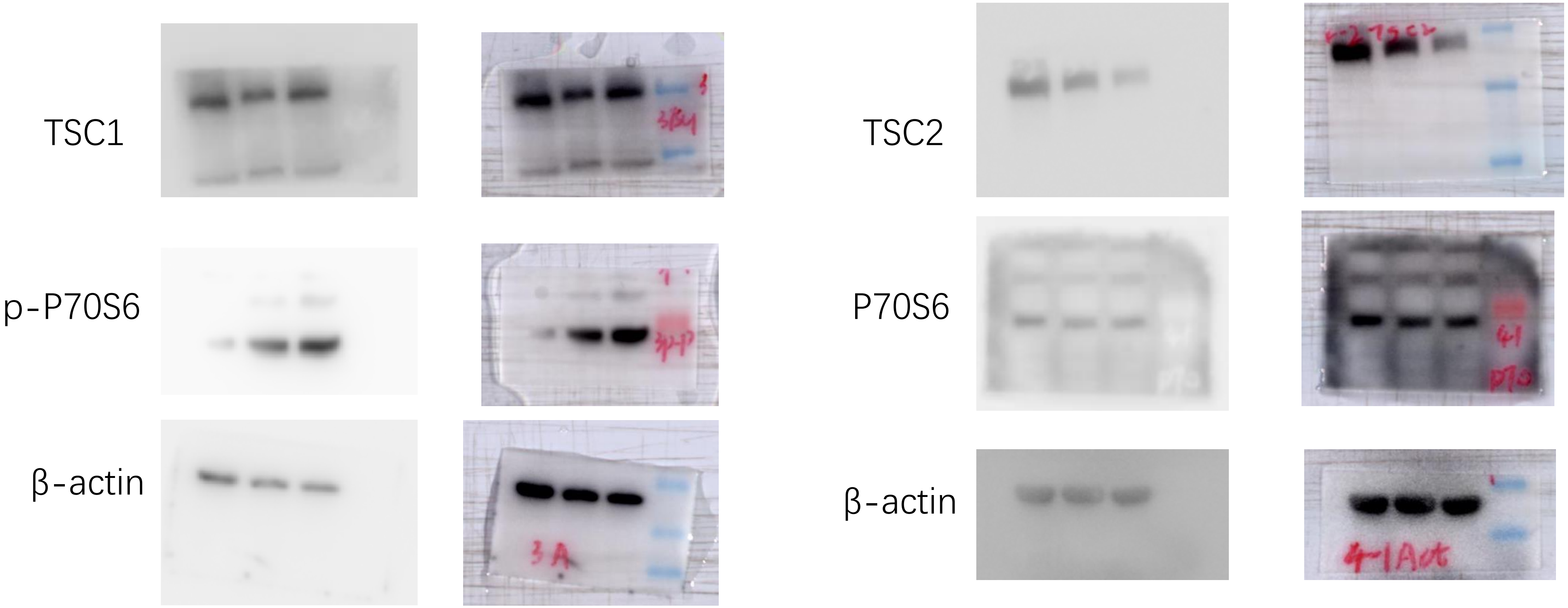

Fig2d-MEF

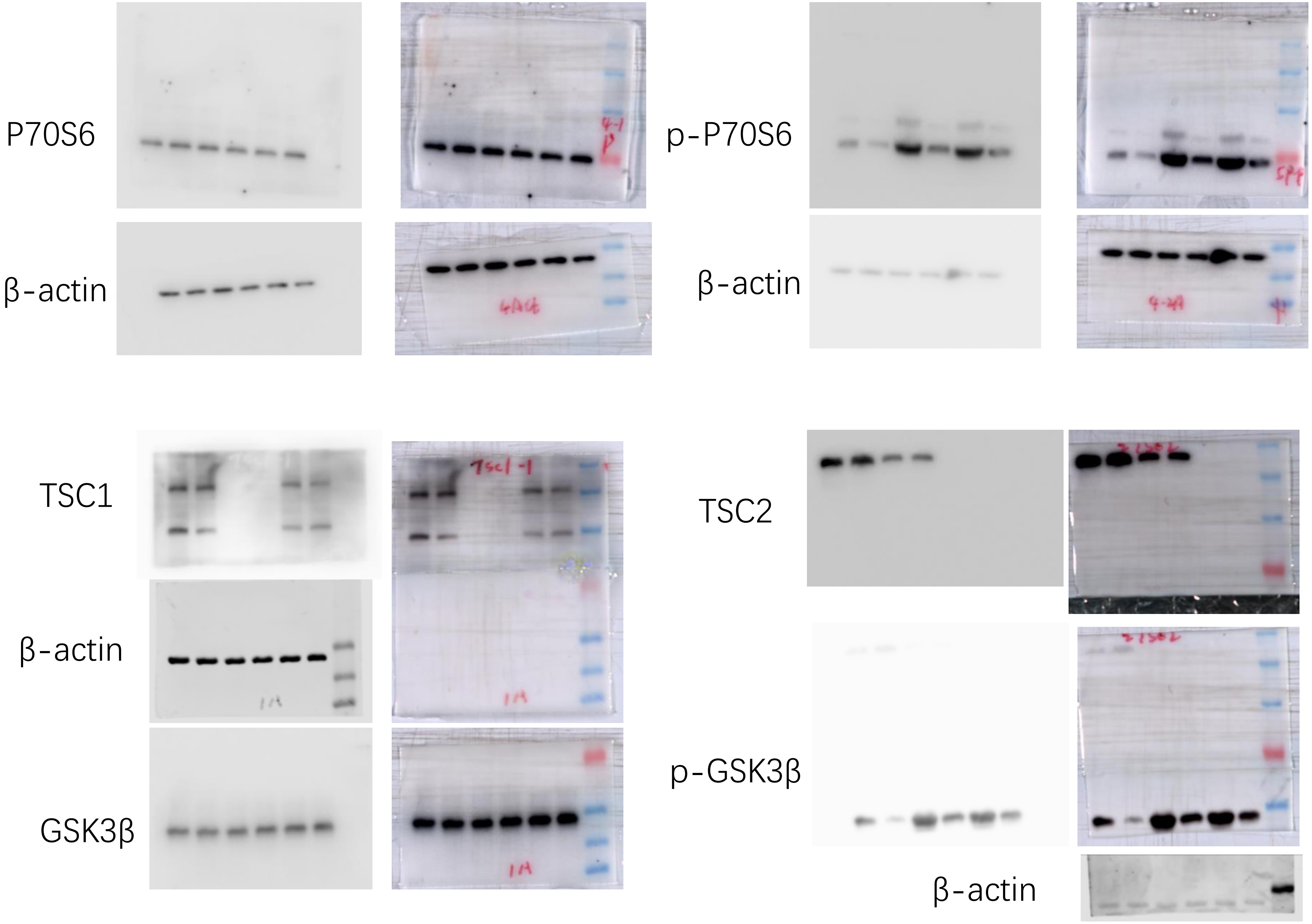
P70S6
β-actin
p-P70S6
β-actin
TSC1
β-actin
GSK3β
TSC2
p-GSK3β
β-actin

Fig3d-MEF

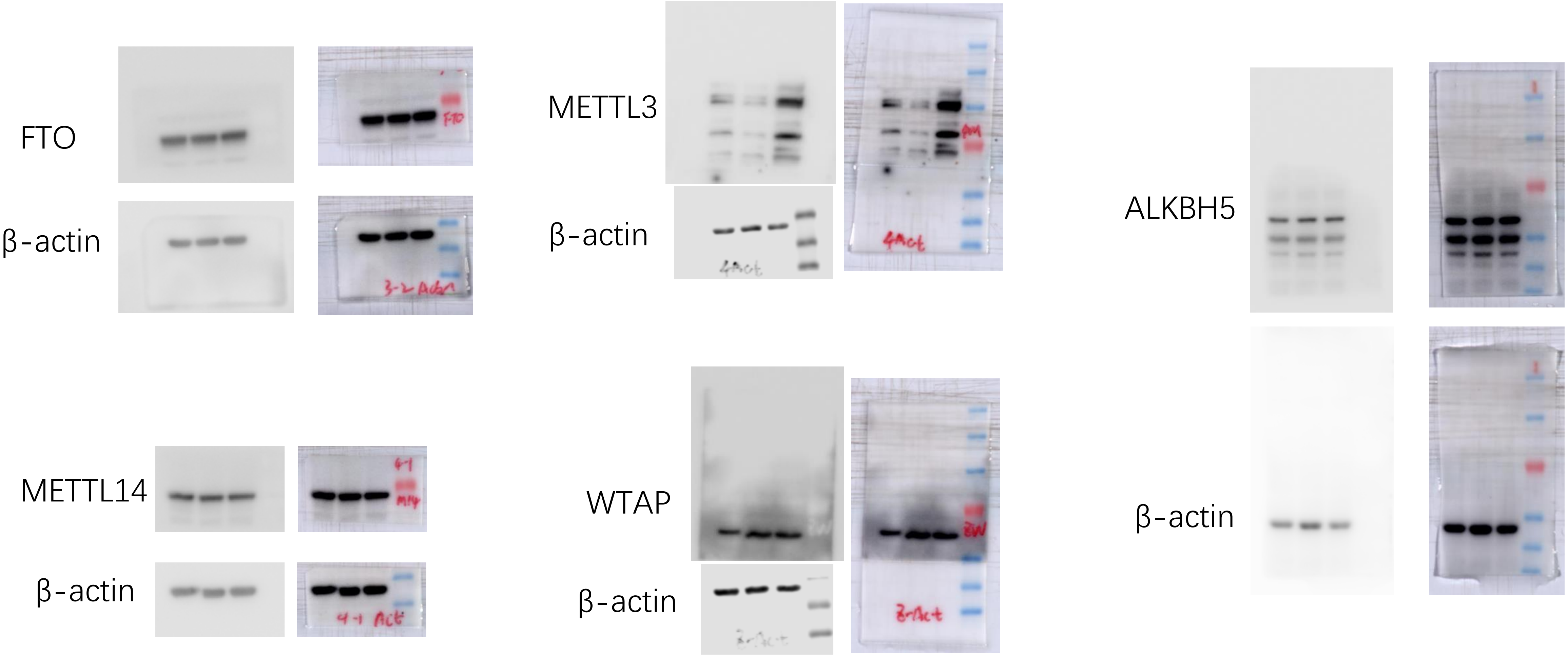
FTO
β-actin
METTL3
β-actin
ALKBH5
β-actin
METTL14
β-actin
WTAP
β-actin

Fig.3d HepG2

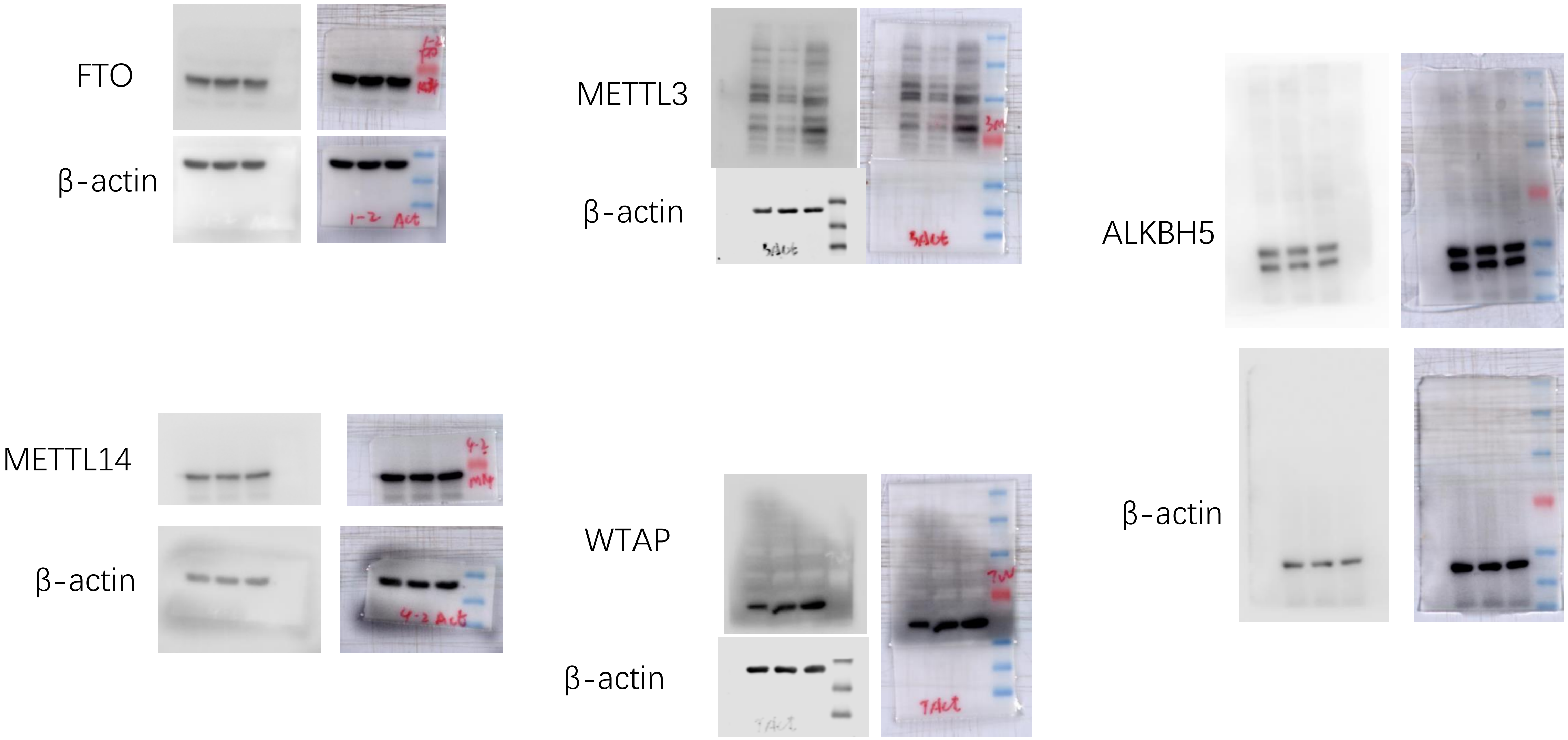
FTO
β-actin
METTL3
β-actin
ALKBH5
β-actin
METTL14
β-actin
WTAP
β-actin

Fig.3e

P70S6

β-actin

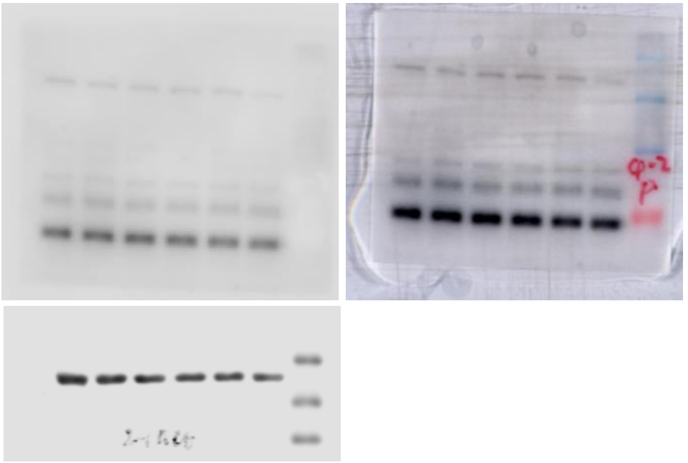

P-P70S6

β-actin

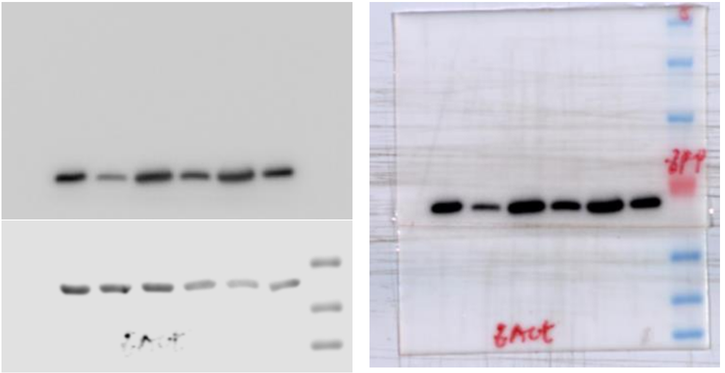

WTAP

β-actin

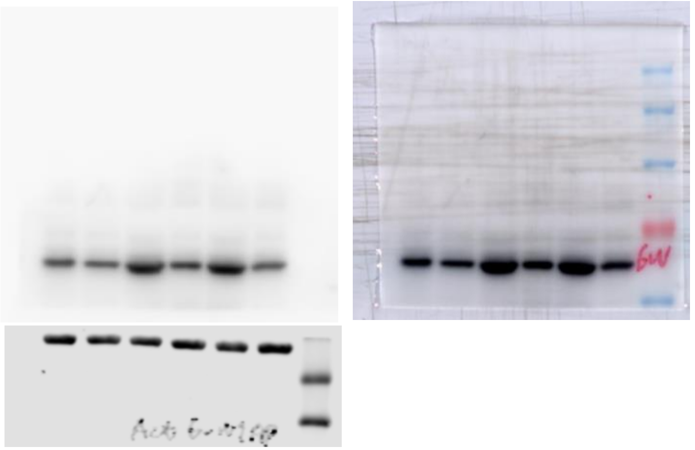

METTL3

β-actin

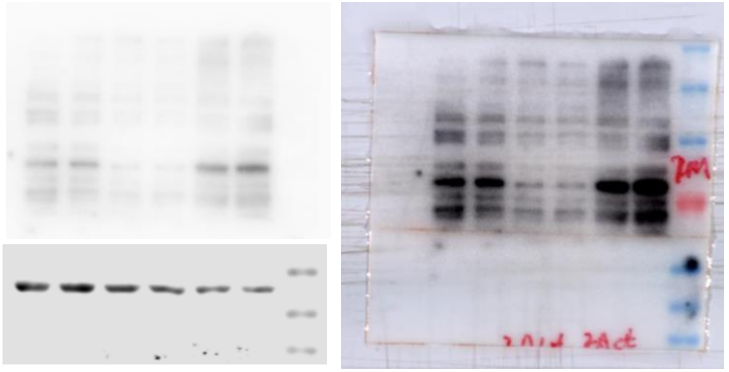

Fig.4a HepG2

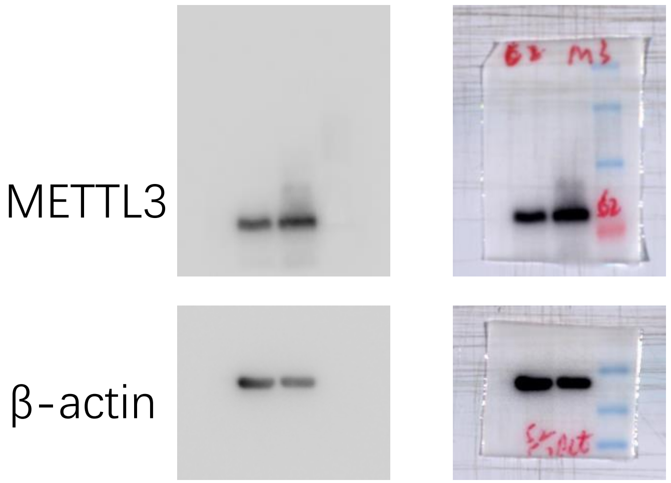

Fig.4d HepG2

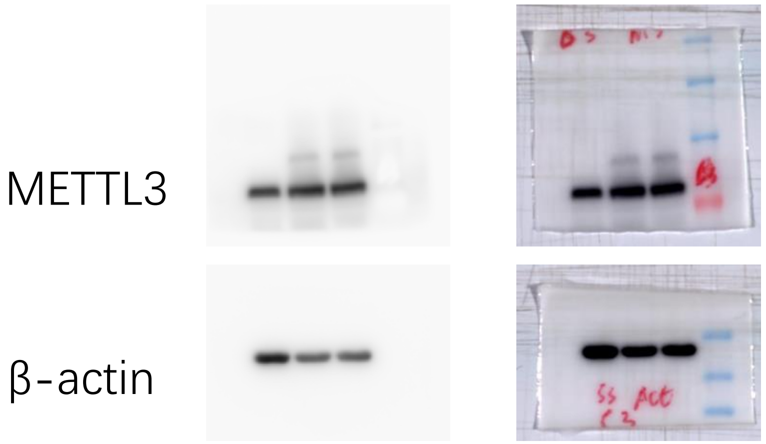

Fig.5a MEF

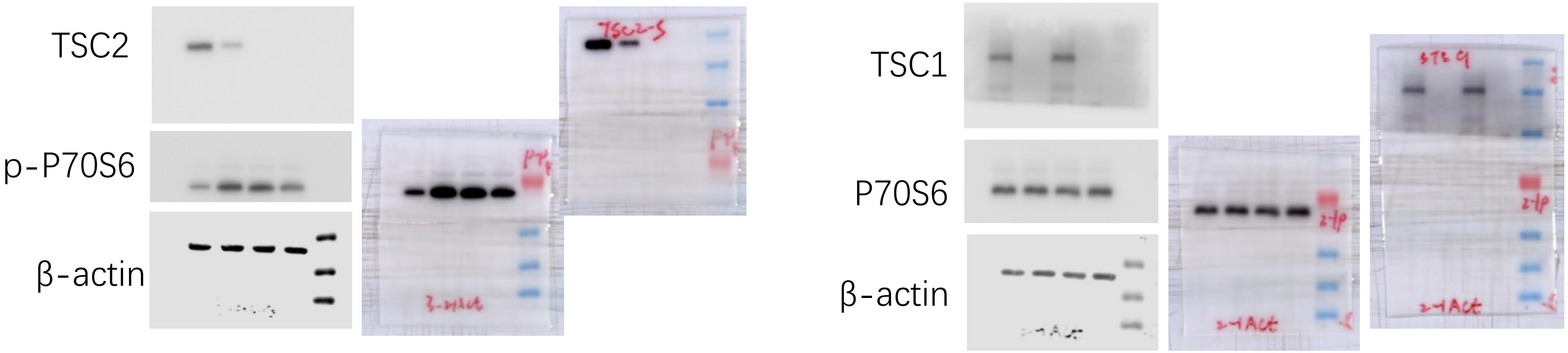

Fig.5a MEF

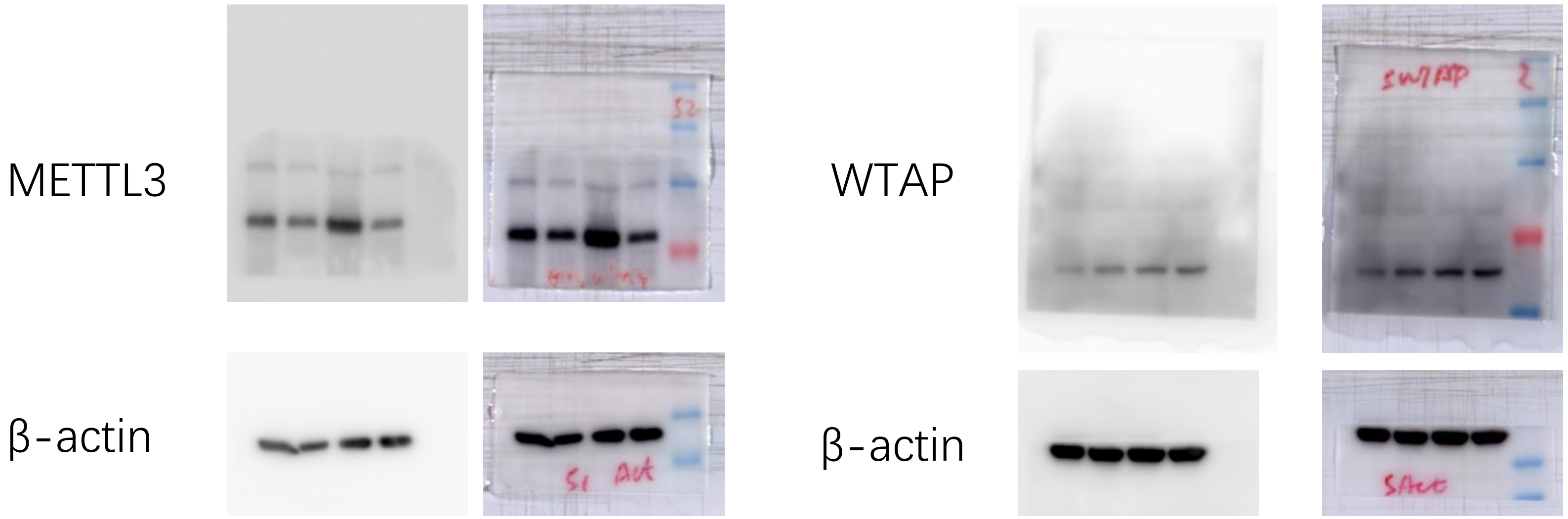

Fig.5a HepG2

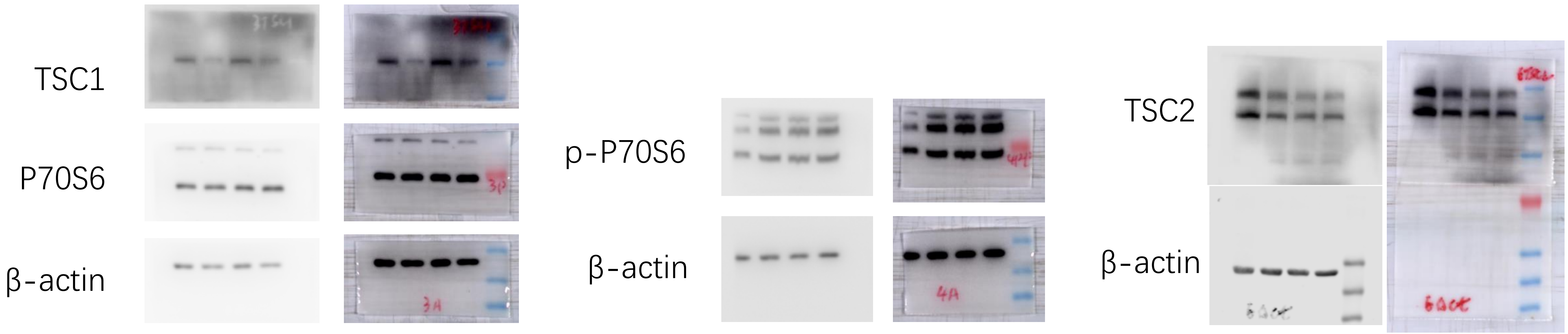

Fig.5a HepG2:

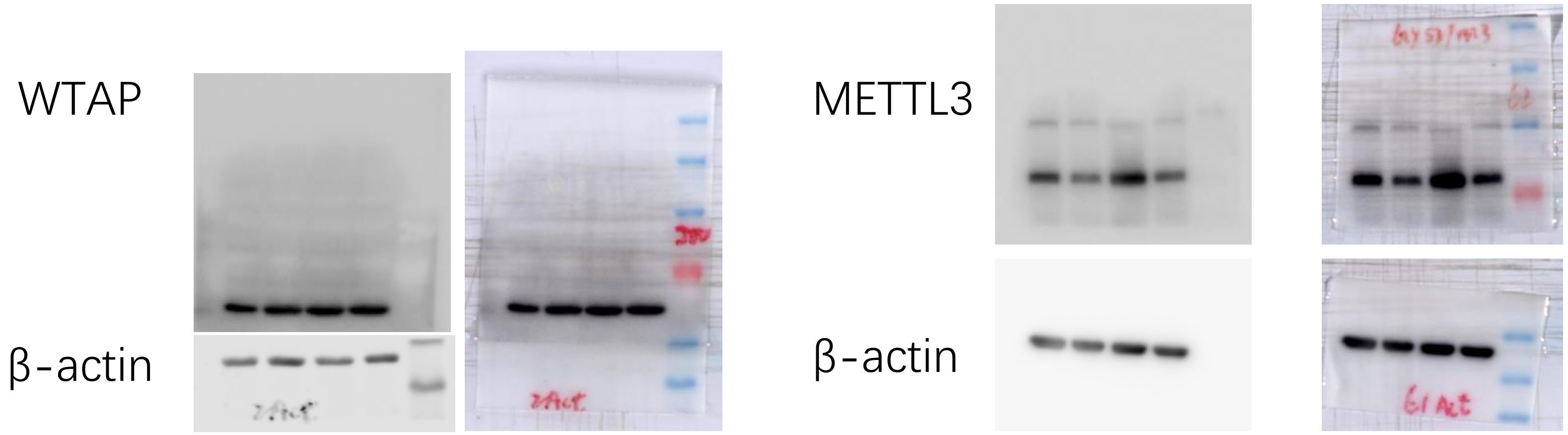

Fig.5b MEF:

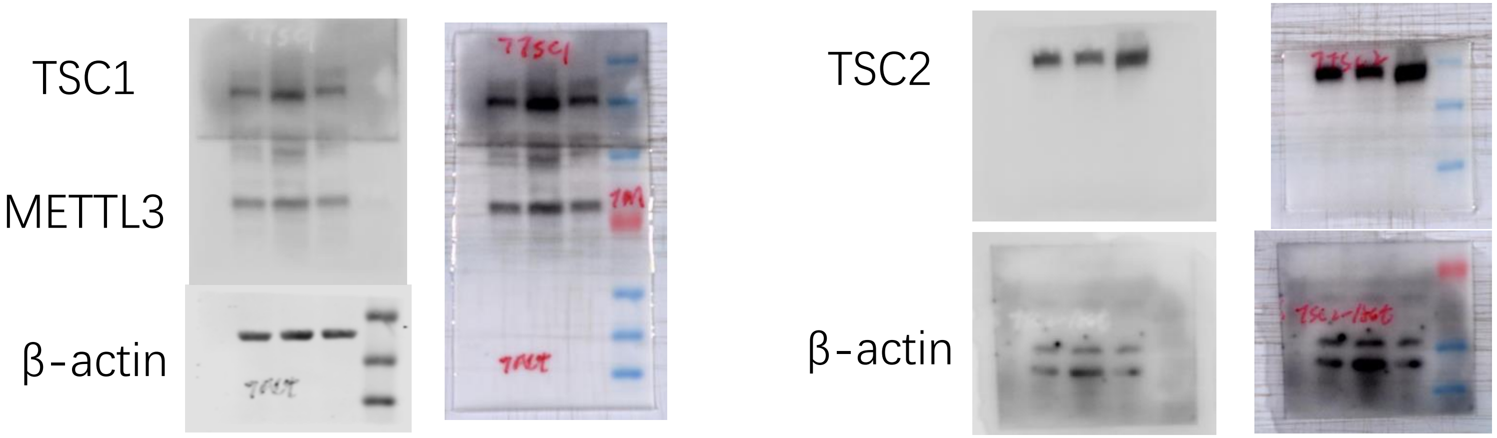

Fig.5b HepG2:

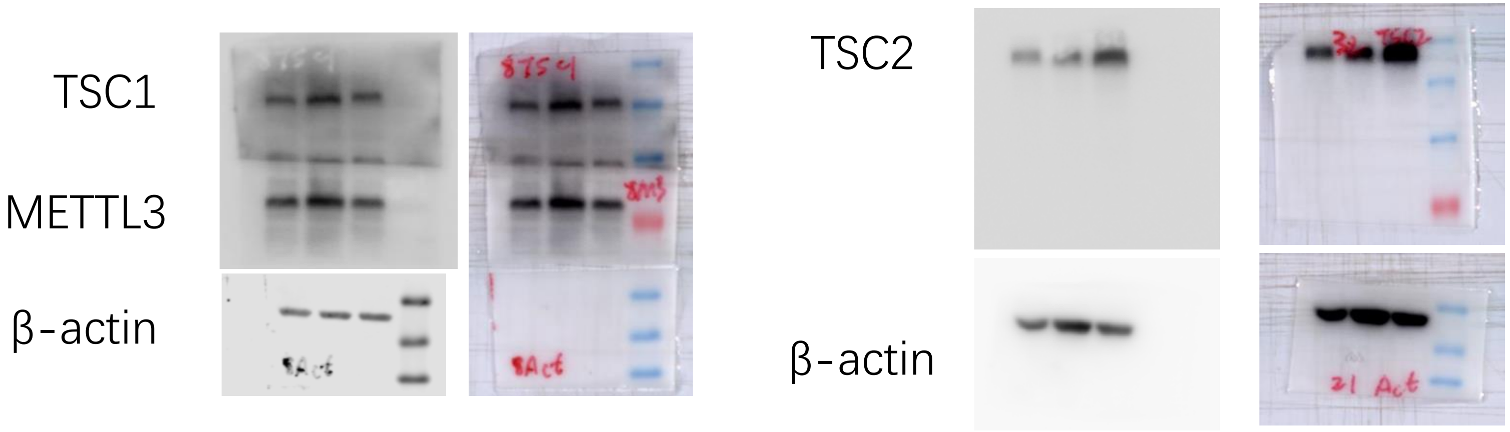

Fig.5d :

TSC2

TSC1

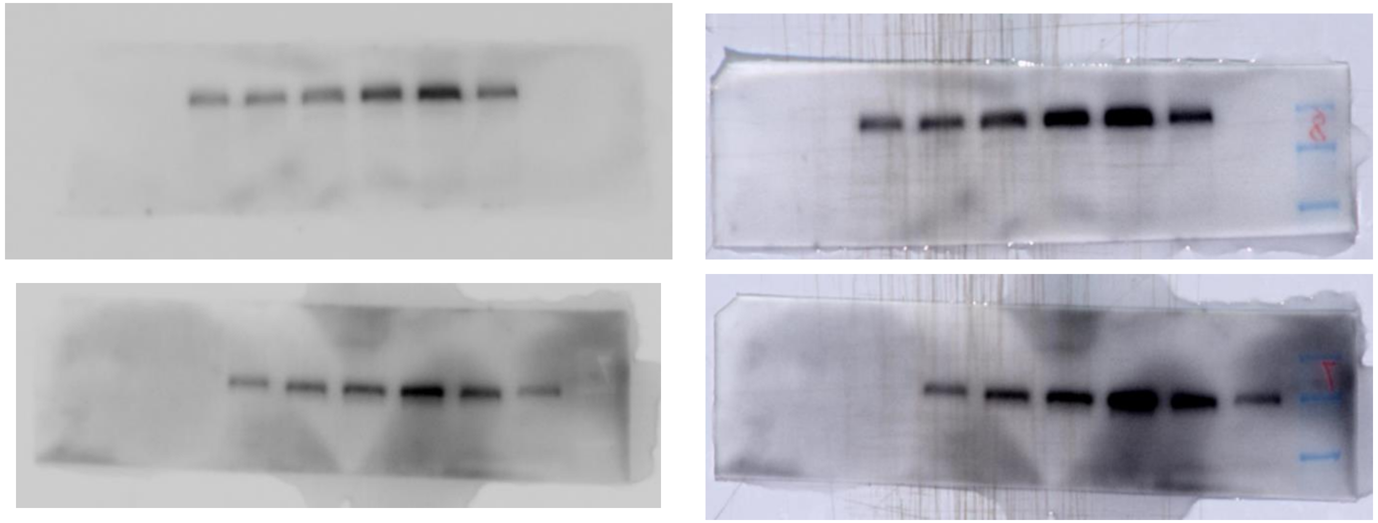

Fig.5e :

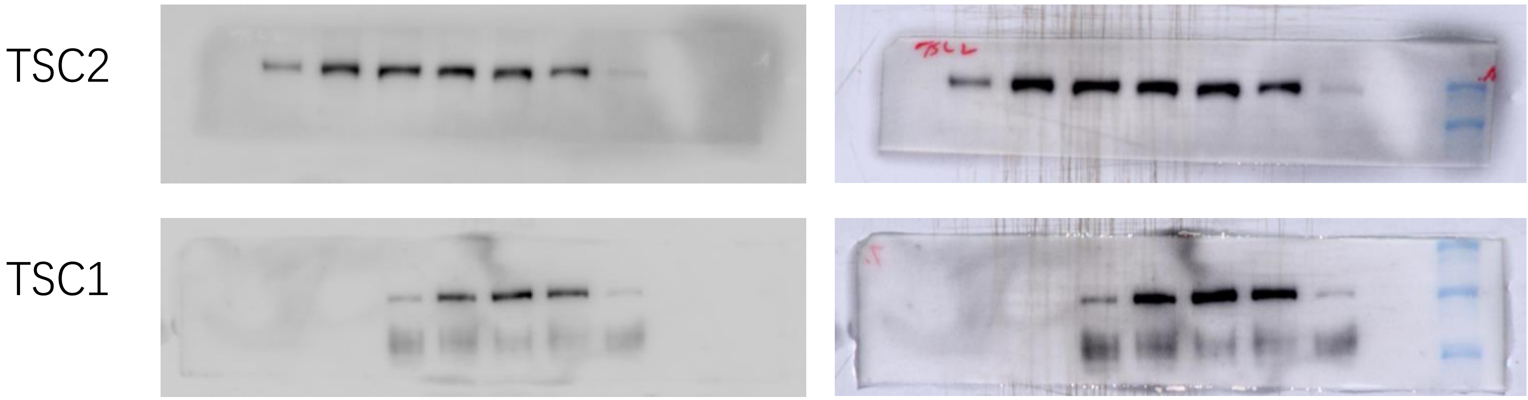

Fig.5f HepG2:

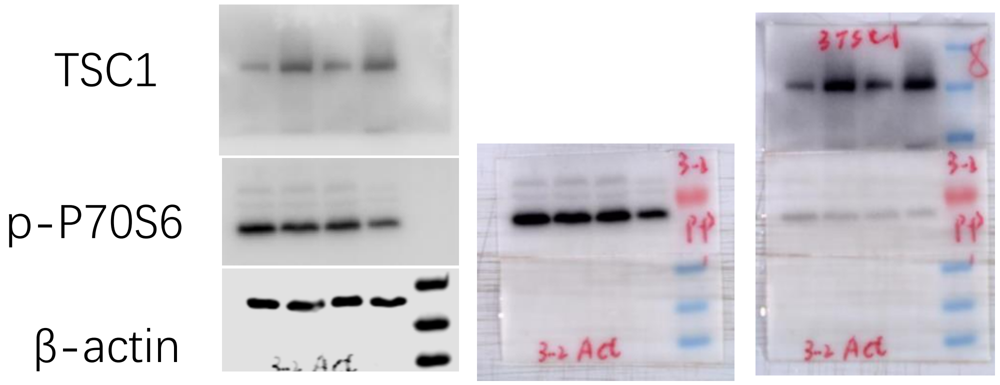

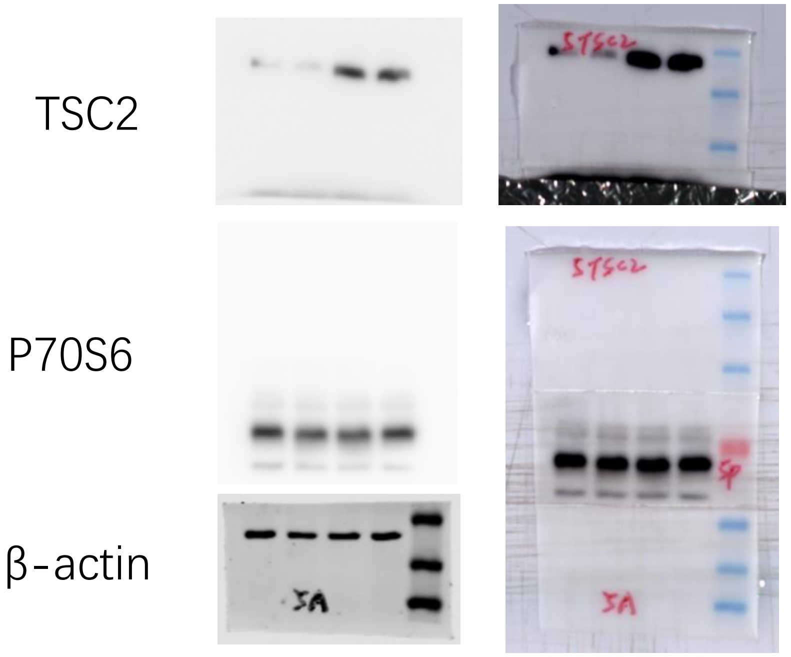

Fig.6d HepG2:

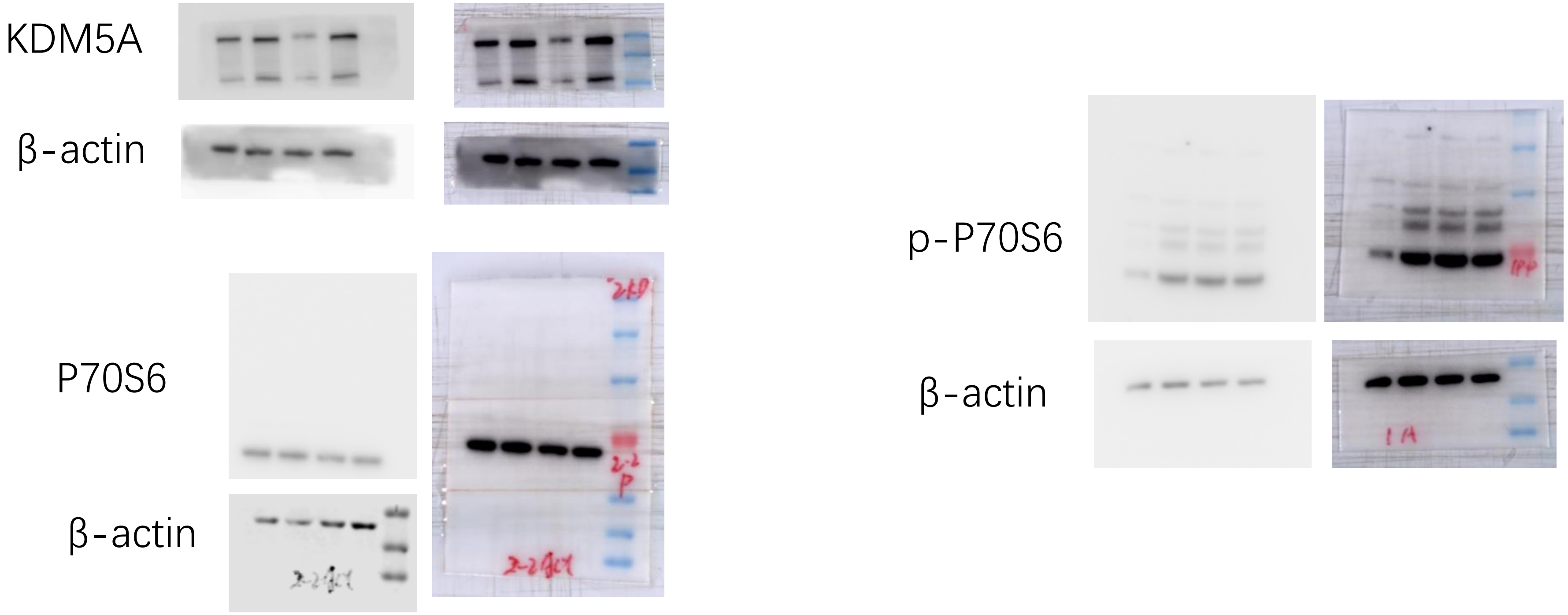

Fig.6e MEF:

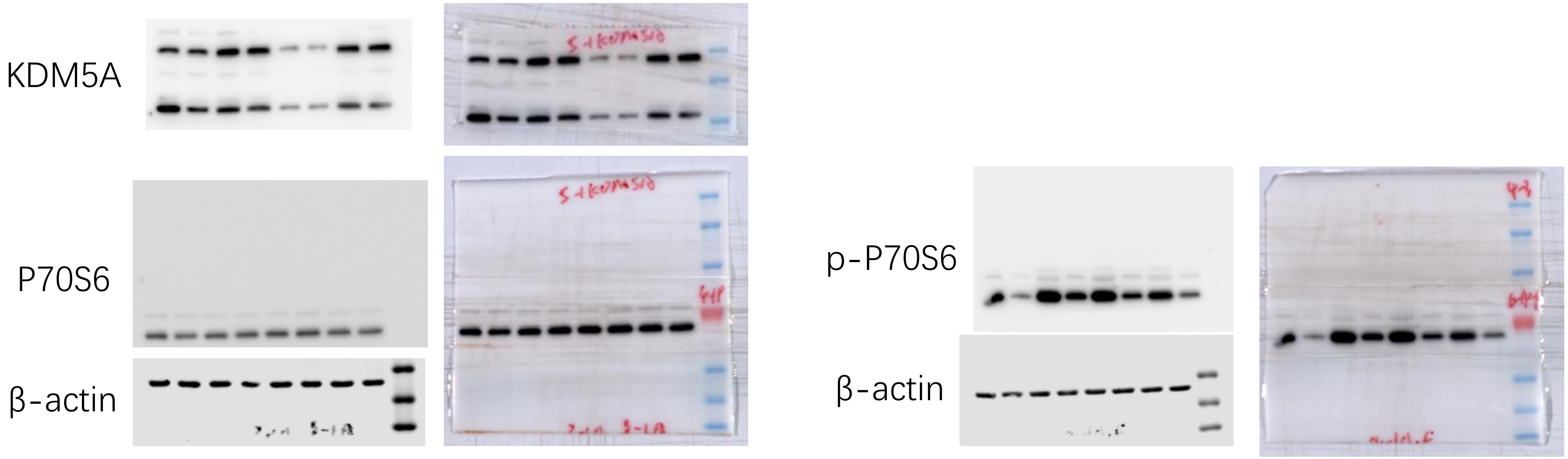

Fig.6f HepG2:

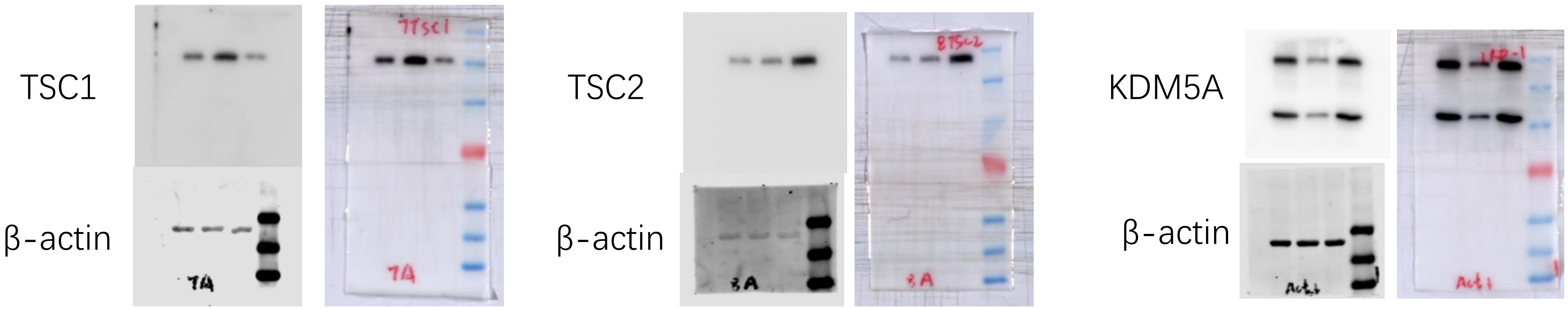

Fig.6g HepG2:

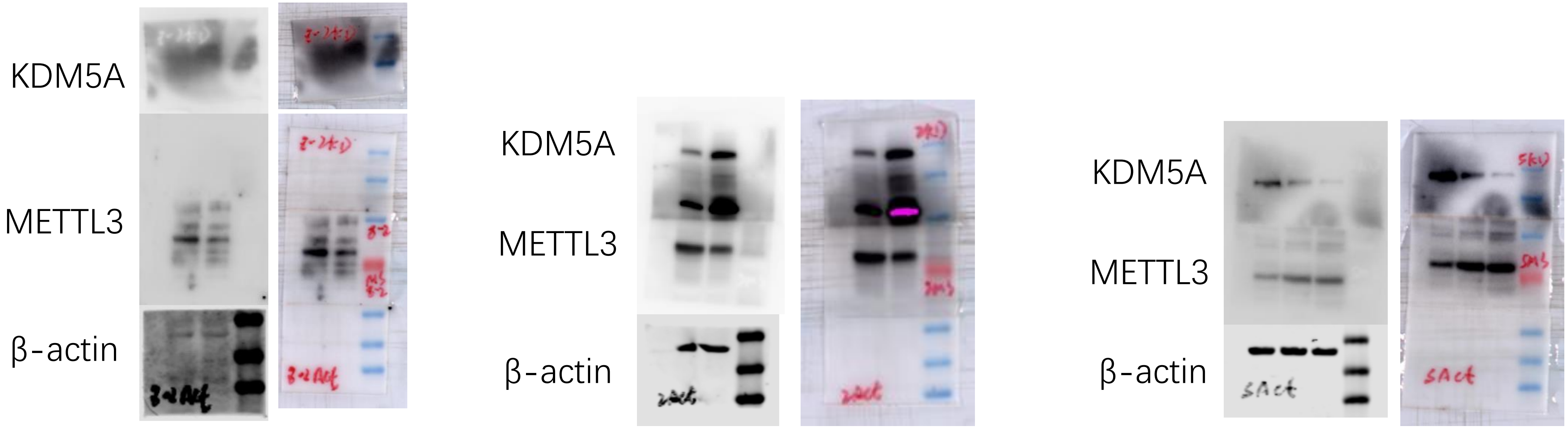
KDM5A
METTL3
β-actin
KDM5A
METTL3
β-actin
KDM5A
METTL3
β-actin

Fig.7a :

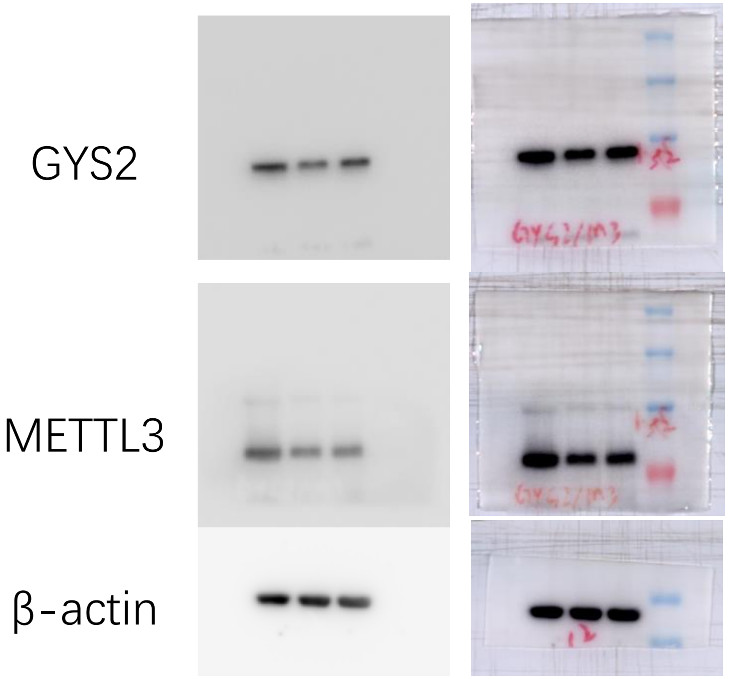

Fig.7b :

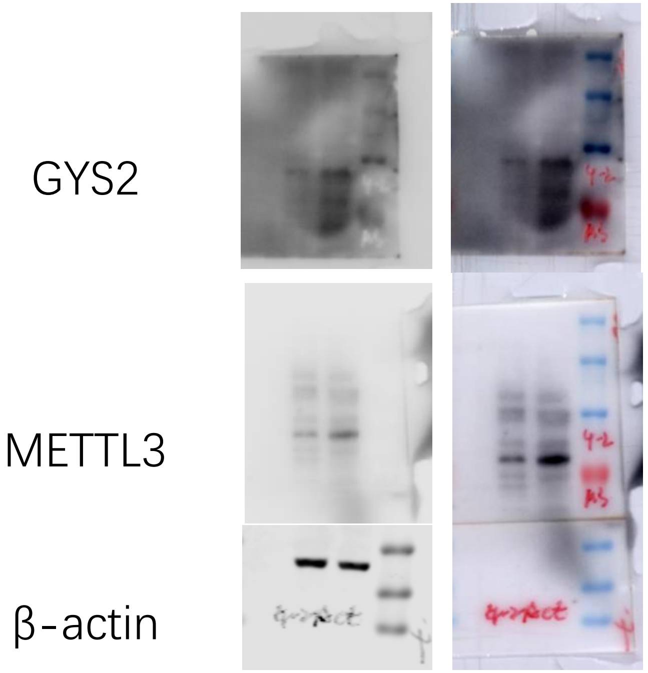

Fig.7c :

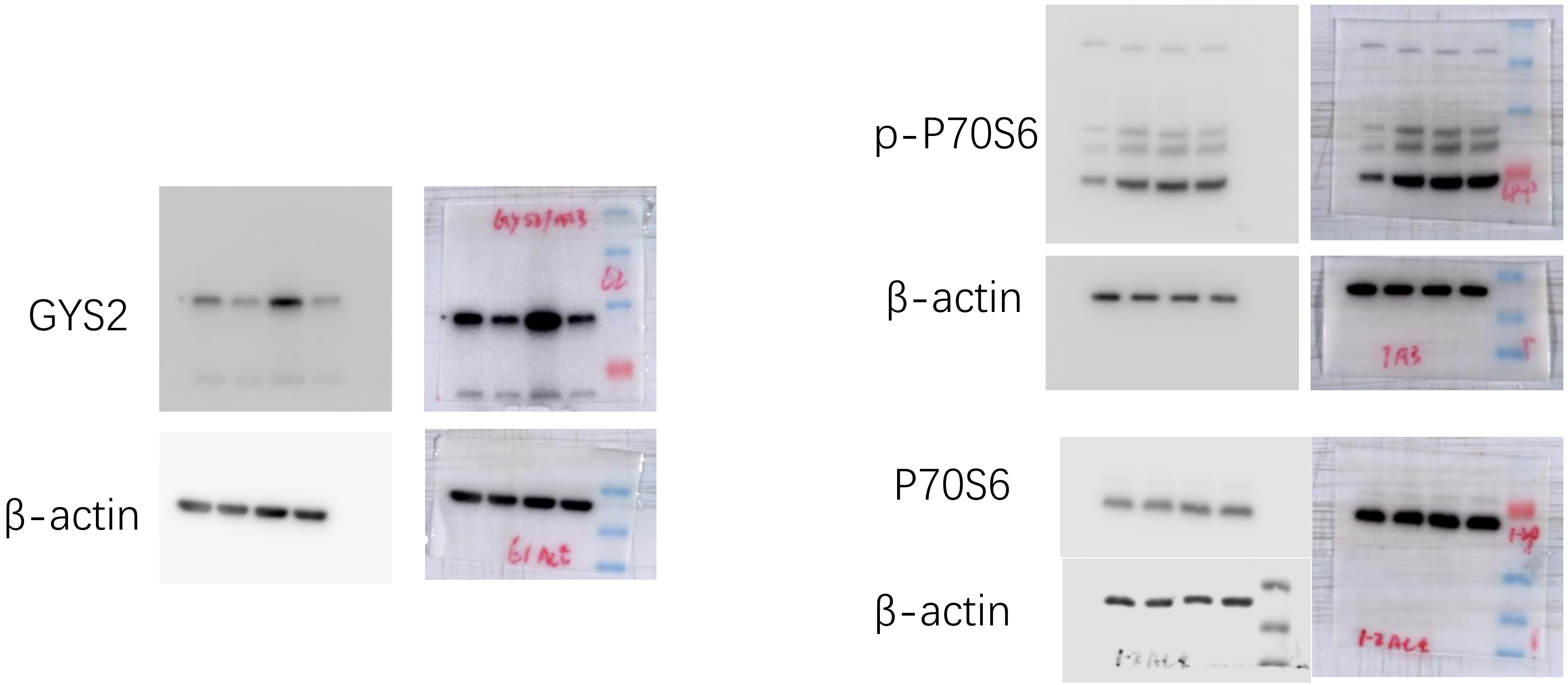

Fig.7d :

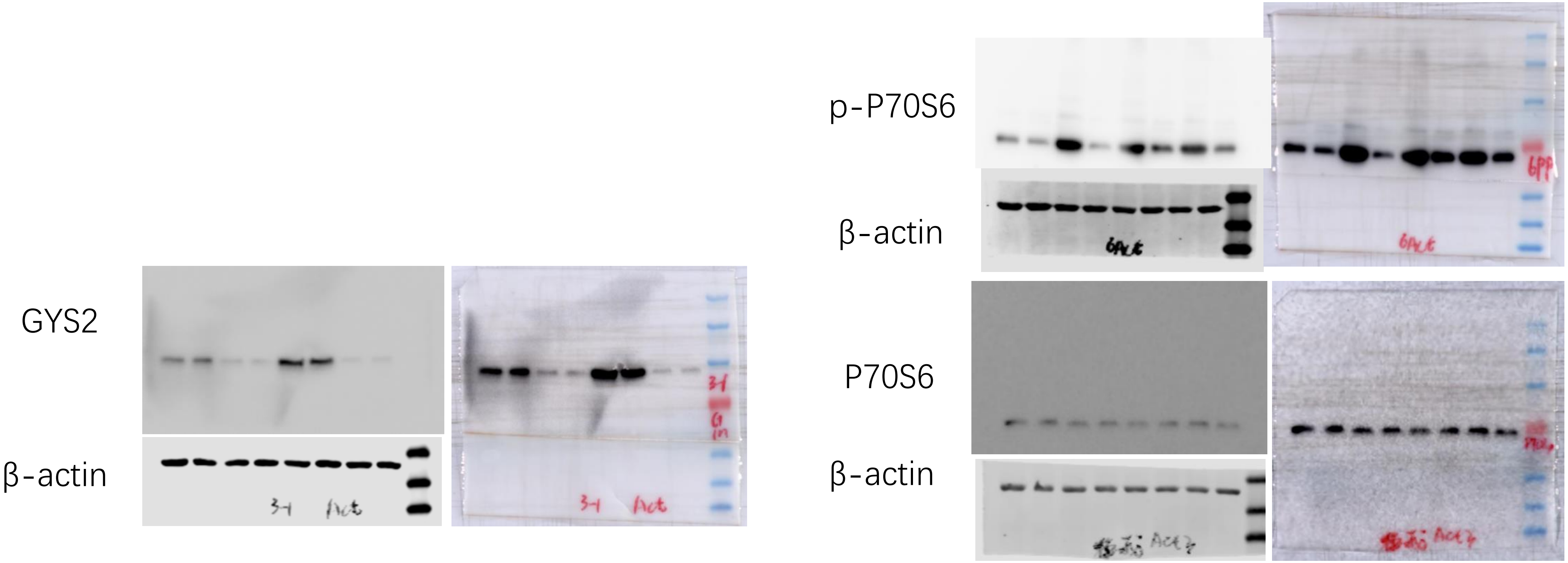

Fig.7e :

TSC2

GYS2

β-actin

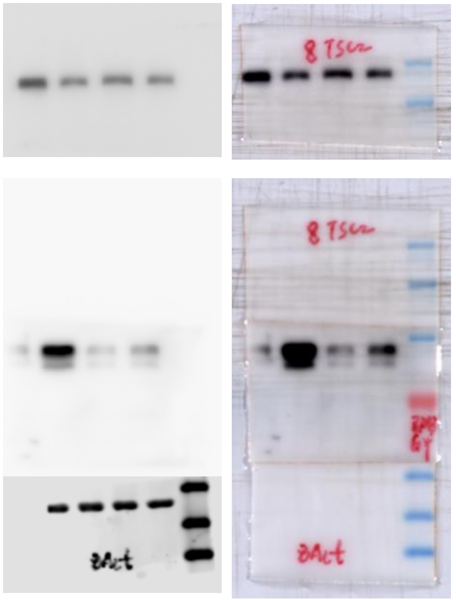

METTL3

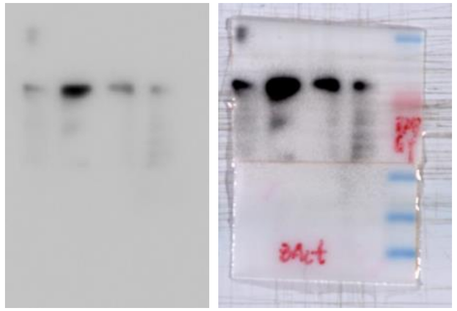

Fig.7f :

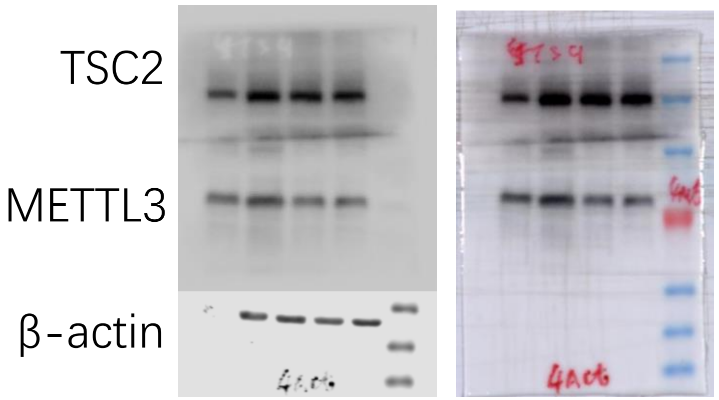

Fig.7k:

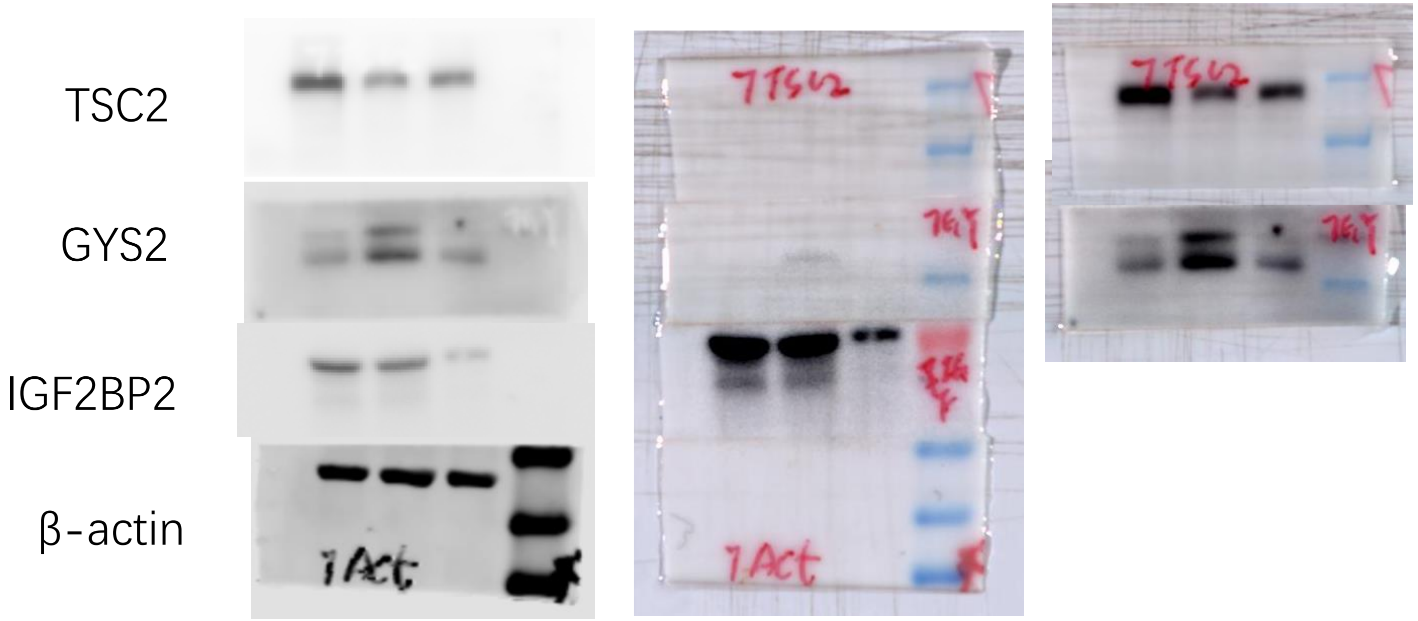

Fig.7m:

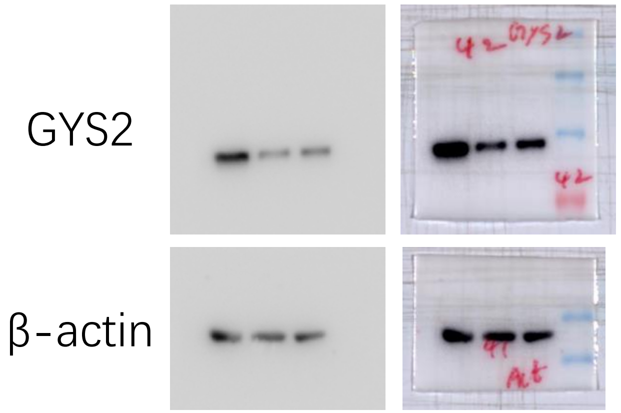

Supplemental Fig.2

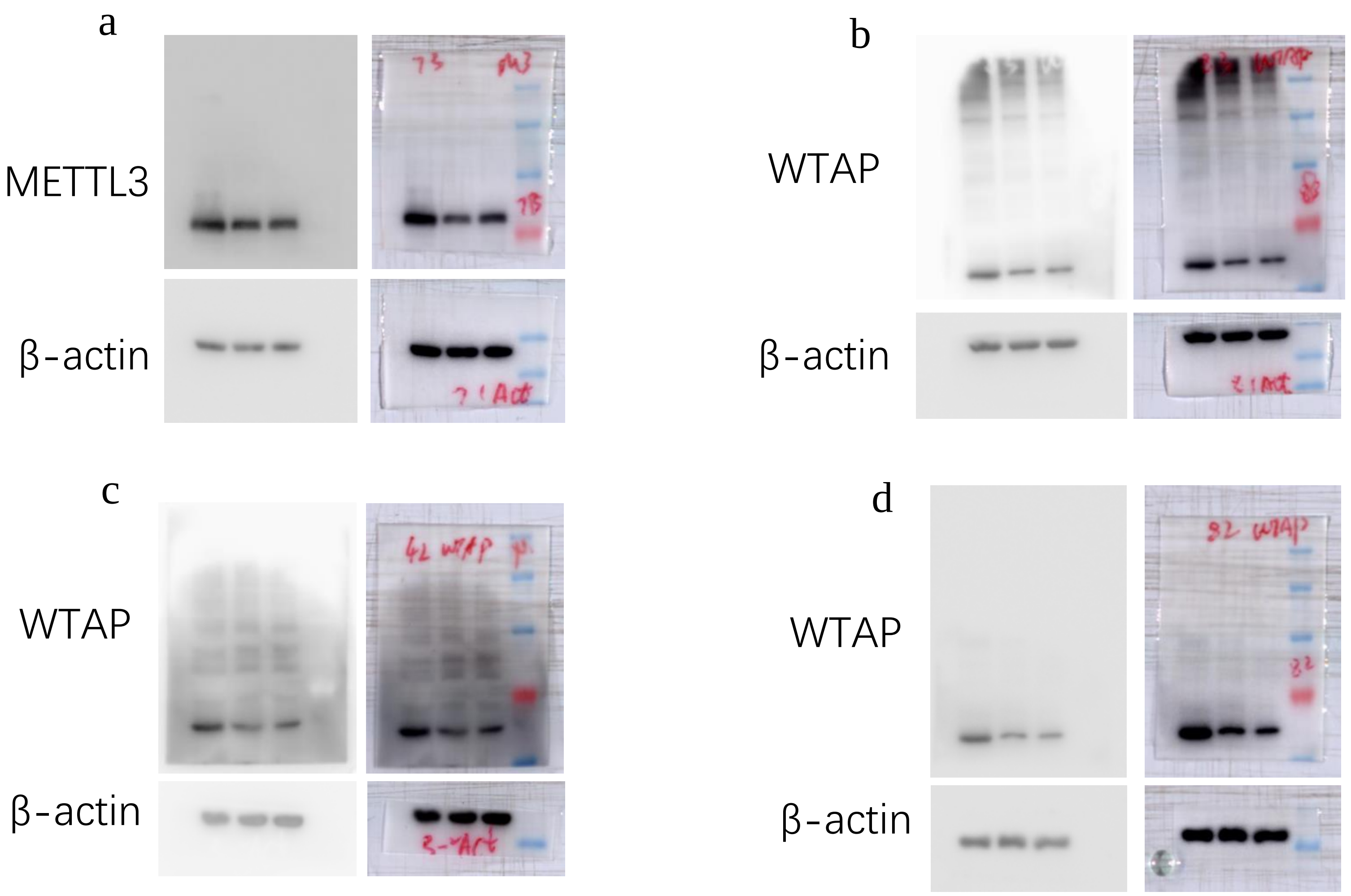

Supplemental Fig.4

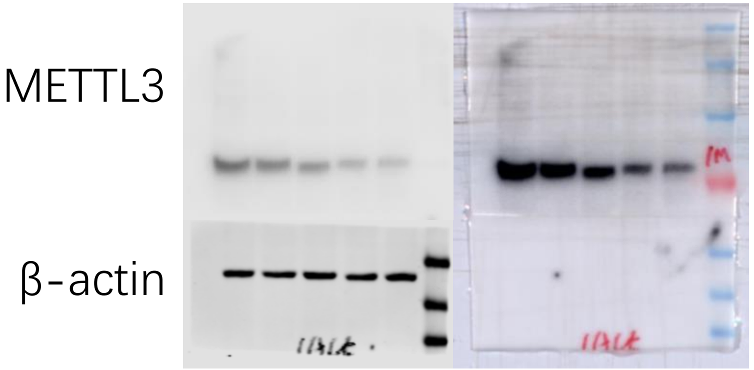

WT MEFs

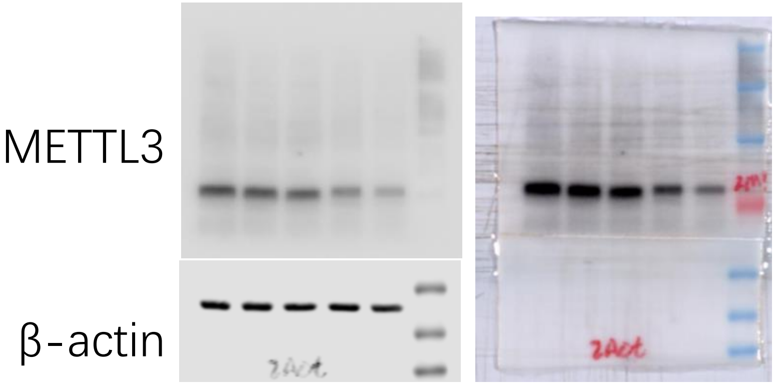

Tsc2$^{-/-}$ MEFs

Supplemental Fig.5

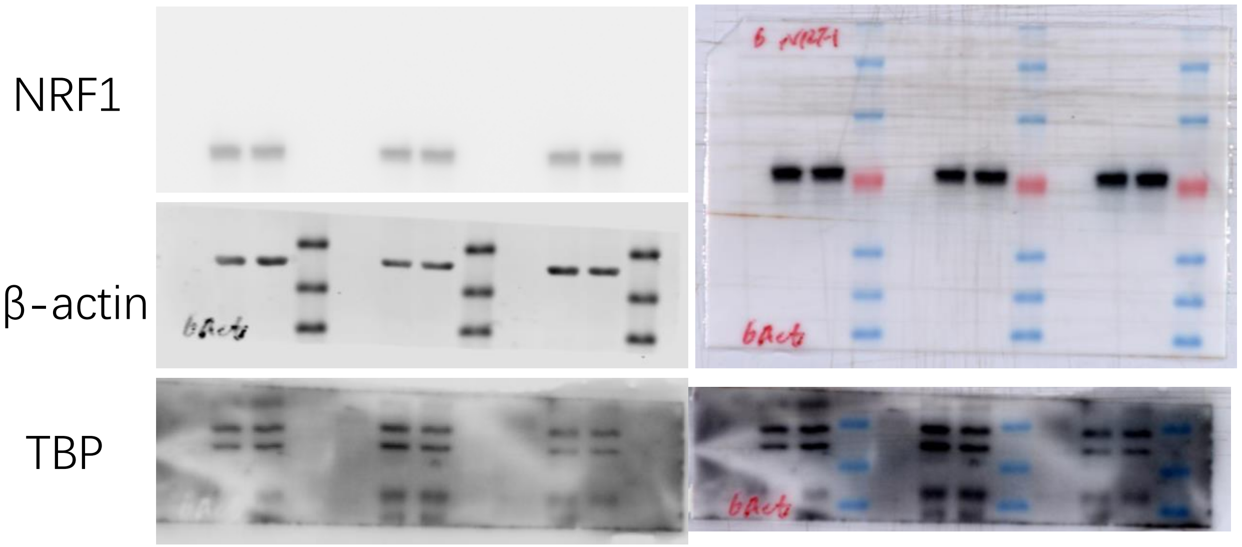

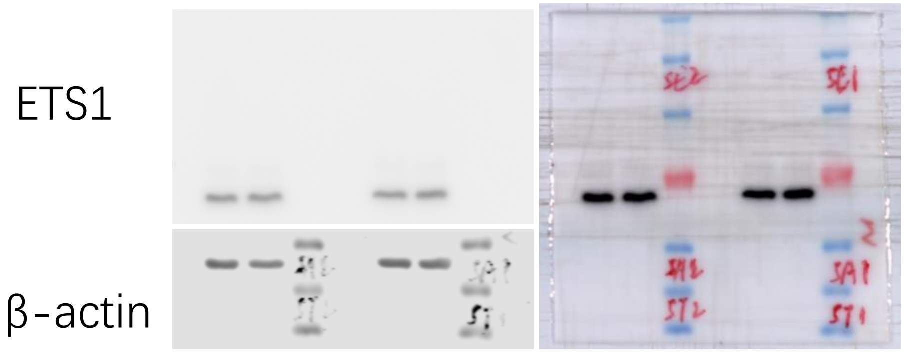

Supplemental Fig.8

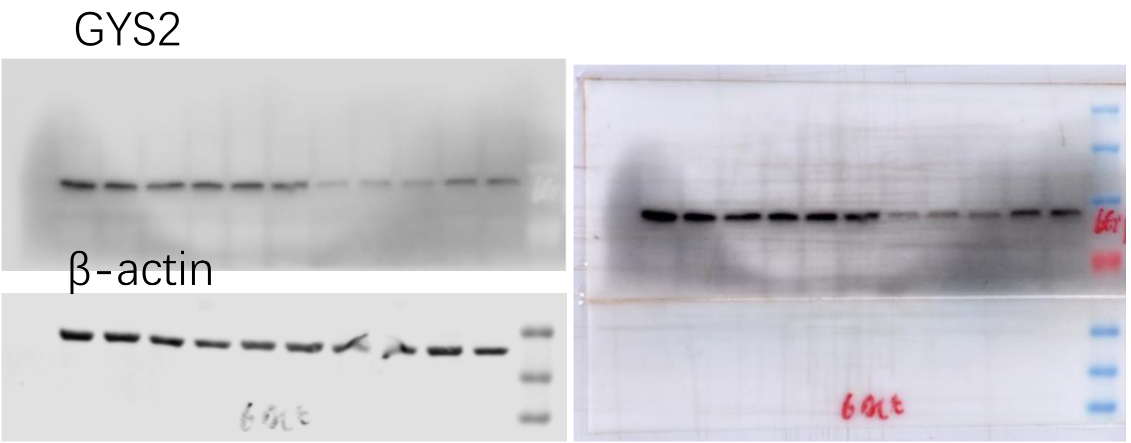

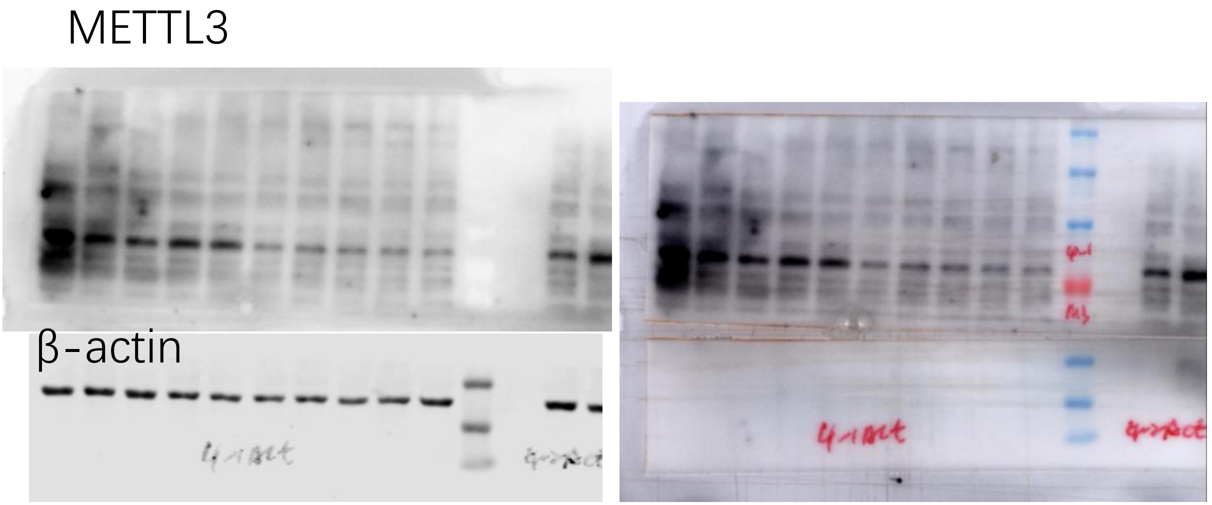

Supplement: Supplementary file 3 — Full and uncropped western blots [file 41419_2025_8161_MOESM3_ESM.pdf]
